# Memory Th1 Cells Are Protective in Invasive *Staphylococcus aureus* Infection

**DOI:** 10.1371/journal.ppat.1005226

**Published:** 2015-11-05

**Authors:** Aisling F. Brown, Alison G. Murphy, Stephen J. Lalor, John M. Leech, Kate M. O’Keeffe, Micheál Mac Aogáin, Dara P. O’Halloran, Keenan A. Lacey, Mehri Tavakol, Claire H. Hearnden, Deirdre Fitzgerald-Hughes, Hilary Humphreys, Jérôme P. Fennell, Willem J. van Wamel, Timothy J. Foster, Joan A. Geoghegan, Ed C. Lavelle, Thomas R. Rogers, Rachel M. McLoughlin

**Affiliations:** 1 Host-Pathogen Interactions Group, School of Biochemistry and Immunology, Trinity Biomedical Sciences Institute, Trinity College Dublin, Dublin, Ireland; 2 Department of Clinical Microbiology, School of Medicine, Trinity College Dublin, Dublin, Ireland; 3 Department of Microbiology, Moyne Institute of Preventive Medicine, School of Genetics and Microbiology, Trinity College Dublin, Dublin, Ireland; 4 Department of Medical Microbiology & Infectious Diseases, Erasmus Medical Center, Rotterdam, Netherlands; 5 Adjuvant Research Group, School of Biochemistry and Immunology, Trinity Biomedical Sciences Institute, Trinity College Dublin, Dublin, Ireland; 6 Department of Clinical Microbiology, Royal College of Surgeons in Ireland, Dublin, Ireland; 7 Department of Microbiology, Beaumont Hospital, Dublin, Ireland; 8 Department of Clinical Microbiology, Adelaide Meath & National Children’s Hospital, Dublin, Ireland; 9 Advanced Materials and Bioengineering Research Centre (AMBER), Centre for Research on Adaptive Nanostructures and Nanodevices (CRANN), Trinity College Dublin, Dublin, Ireland; 10 Department of Clinical Microbiology, St. James's Hospital, Dublin, Ireland; Johns Hopkins School of Medicine, UNITED STATES

## Abstract

Mechanisms of protective immunity to *Staphylococcus aureus* infection in humans remain elusive. While the importance of cellular immunity has been shown in mice, T cell responses in humans have not been characterised. Using a murine model of recurrent *S*. *aureus* peritonitis, we demonstrated that prior exposure to *S*. *aureus* enhanced IFNγ responses upon subsequent infection, while adoptive transfer of *S*. *aureus* antigen-specific Th1 cells was protective in naïve mice. Translating these findings, we found that *S*. *aureus* antigen-specific Th1 cells were also significantly expanded during human *S*. *aureus* bloodstream infection (BSI). These Th1 cells were CD45RO^+^, indicative of a memory phenotype. Thus, exposure to *S*. *aureus* induces memory Th1 cells in mice and humans, identifying Th1 cells as potential *S*. *aureus* vaccine targets. Consequently, we developed a model vaccine comprising staphylococcal clumping factor A, which we demonstrate to be an effective human T cell antigen, combined with the Th1-driving adjuvant CpG. This novel Th1-inducing vaccine conferred significant protection during *S*. *aureus* infection in mice. This study notably advances our understanding of *S*. *aureus* cellular immunity, and demonstrates for the first time that a correlate of *S*. *aureus* protective immunity identified in mice may be relevant in humans.

## Author Summary

*Staphylococcus aureus* is a leading cause of skin, soft tissue and bone infections and, most seriously, bloodstream infection. When *S*. *aureus* does get into the bloodstream, it is more likely to kill than any other bacterial infection, despite all the treatments modern medicine has to offer. It has thus far developed resistance to all antibiotics licensed to treat it. Thus, there is an urgent need to develop a vaccine against *S*. *aureus*. However, such a vaccine remains elusive. This is largely due to the fact that we have a very limited understanding of how our immune system fights this infection. Here, we examine how certain T cells of the mouse immune system effectively recognise and respond to *S*. *aureus*, and show that transferring these cells to other mice improves their ability to clear infection. We then demonstrate that a vaccine which drives these specific T cells also improves clearance of infection. Until recently, it was not known if human T cells could recognise and respond to *S*. *aureus*. Here we show, for the first time, that these cells are expanded in patients with *S*. *aureus* bloodstream infection, and suggest that they should be targeted in anti-*S*. *aureus* vaccines.

## Introduction

*Staphylococcus aureus* is a leading cause of community- and hospital-acquired bacterial infections. It is one of the most common causes of bloodstream infection (BSI), and carries a higher mortality than any other bacteraemia (20–40% within 30 days) despite appropriate treatment [1]. It is also the leading cause of other serious infections including osteomyelitis, septic arthritis, endocarditis and device-related infections, and leads to significant healthcare costs [2]. The burden of *S*. *aureus* disease is amplified by the fact that resistance has been demonstrated to every licensed anti-staphylococcal agent to date [3]. Consequently, there is an urgent unmet clinical need to develop a vaccine against *S*. *aureus*.

Several model vaccines have shown an ability to prevent or attenuate *S*. *aureus* infection in murine models, but no candidate vaccines have yet shown efficacy in human clinical trials. Progress towards an efficacious *S*. *aureus* vaccine has been significantly compromised because murine models do not sufficiently recapitulate human exposure to *S*. *aureus* [4]. Mice are not natural hosts for *S*. *aureus*–they require high bacterial inocula to establish systemic disease and, critically, are immunologically naïve on first infection. In contrast, humans are imprinted with an immune response following multiple exposures to *S*. *aureus*, and its successful commensalism facilitates an intimate association with the host immune system that has enabled the evolution of multiple bacterial mechanisms to evade innate and adaptive immunity [5]. *S*. *aureus* vaccine development is significantly impeded by a fundamental lack of understanding of the correlates of immune protection in humans, and our knowledge of which elements of the immune response are important in recovery from or prevention of human *S*. *aureus* infection is extremely limited.

Antibody responses to *S*. *aureus* antigens have traditionally been used as biomarkers for vaccine immunogenicity. However, vaccines that have produced robust humoral immunity have not prevented or attenuated the course of infection in clinical trials, nor has passive immunisation [6]. This is possibly to be expected as it is unclear whether B cell deficiency states in humans or in mice result in greater incidence or severity of invasive *S*. *aureus* disease [6–8]. Given that *S*. *aureus* persistently or transiently colonises most of the population, human serum has a pre-existing and variable repertoire of anti-*S*. *aureus* antibodies [9]. Limited clinical data suggests that higher levels of anti-toxin antibodies may attenuate disease in *S*. *aureus* BSI [10]. However, it has proven difficult to consistently correlate the presence or titre of anti-staphylococcal antibodies with improved clinical outcomes [11,12]. On the other hand, defects in cellular immunity–both in mice and in humans–are reliably associated with increased risk of *S*. *aureus* infection.

There is accumulating evidence that T helper (Th) cells play important roles in protection against human *S*. *aureus* infection [13]. Underlying conditions such as cancer, HIV infection, end-stage renal disease and diabetes mellitus present the greatest relative risk for *S*. *aureus* BSI acquisition, and these pathologies are all associated with impaired Th1 cell responses [14–17]. Patients with the rare autosomal dominant hyper-IgE syndrome (AD-HIES) are also prone to recurrent staphylococcal skin and lung abscesses [18]. In these patients, mutations in STAT3 (signal transducer and activator of transcription 3) result in impaired Th17 cell development [18], while CD4^+^ cells retain the ability to differentiate into other subsets [19]. Interestingly, these patients do not seem more prone to *S*. *aureus* bloodstream infection, suggesting that a Th17 response is critically important at skin and respiratory sites only [20]. Other cohorts who present with life-threatening and recurrent *S*. *aureus* infections retain normal T helper cell activity, but instead exhibit defects in phagocyte function [21]. Phagocytes from patients with chronic granulomatous disease (CGD), for example, are unable to generate reactive oxygen species (ROS), which markedly impairs their ability to eliminate certain pathogens, including *S*. *aureus* [22]. Overall, clinical observations suggest that defects in either T helper cell or phagocyte function are associated with increased susceptibility to *S*. *aureus* infection. Importantly, these cell populations are intricately linked. T cells do not directly kill bacteria but, via cytokines such as IFNγ and IL-17, they can orchestrate many downstream effects on phagocytes that enhance their microbicidal activity [23,24].

Murine studies have illuminated the protective roles played by individual T cell subsets in *S*. *aureus* infection, although there have been some contrasting reports on their importance [25]. It is likely that the nature of the infecting stain [26], and the site of infection significantly impact upon the nature of the T cell response elicited [27,28]. T helper cells are crucial to survival following experimental intravenous *S*. *aureus* inoculation [8], and a protective role for Th1 cells is suggested by the finding that IFNγ-deficient mice are hypersusceptible to such infection [29]. IL-17 has also been identified as an important correlate of immune protection. We have previously demonstrated that IL-17-producing γδ^+^ T cells were protective in a peritonitis model of systemic *S*. *aureus* infection [30]. Mice deficient in IL-17A and IL-17F had increased susceptibility to opportunistic mucocutaneous *S*. *aureus* infection compared to wild type mice, but no difference in pathology was observed following systemic infection [31]. This mucocutaneous site-specific protective role for IL-17 during *S*. *aureus* infection parallels the clinical picture in patients with IL-17 deficiencies [20].

Evidence from animal models, in conjunction with clinical observations in patients particularly susceptible to *S*. *aureus* infection, implies that an intact pathway of *S*. *aureus*-responsive T cells mediating phagocyte activation via pro-inflammatory cytokine signalling (IFNγ ± IL-17) is essential to *S*. *aureus* immunity. Accordingly, model vaccines based on this premise of cell-mediated immunity have succeeded in generating antibody-independent protection against systemic infection in mice, providing there is sufficient activation of effector T cell subsets [8,32]. Th1 and Th17 cells seem necessary to mediate protection in certain vaccines, and achieve this by enhancing phagocyte recruitment and activation [29,33]. Consequently, T cell subsets are now being investigated as potential targets for human immunisation. Surprisingly, in the case of previously studied anti-*S*. *aureus* vaccines, cellular immune responses were not assessed prior to entering clinical trials and preclinical findings demonstrating the importance of cell-mediated immunity have thus far not been translated into human studies [4]. This has surely contributed to the failure of previous vaccines–especially in light of recent findings which suggest that the majority of adults possess significant levels of circulating antigen-specific memory T cells, indicative of their prior exposures to this organism [34]. A more comprehensive understanding of the role played by specific T cell subsets in site-specific clearance of infection is urgently required to inform development of vaccines that can efficiently promote protective immunity to *S*. *aureus*.

Here, we examine the development and function of *S*. *aureus* antigen-specific T helper cells in mice and, for the first time, translate these findings to humans by profiling memory T cell responses during *S*. *aureus* bloodstream infection. We show that exposure to *S*. *aureus*, in both mice and humans, critically imprints a Th1 cell response. In mice, antigen-specific Th1 cells conferred protection, in part, by promoting macrophage activation–identifying an underappreciated role for these phagocytes in facilitating *S*. *aureus* clearance. Finally, we generate a novel Th1-inducing *S*. *aureus* vaccine comprised of a *S*. *aureus* antigen with the capacity to activate human Th1 cells combined with a potent Th1-inducing adjuvant, and demonstrate the effectiveness of this vaccine in conferring Th1-mediated protective immunity during systemic *S*. *aureus* infection in mice.

## Results

### Prior exposure to *S*. *aureus* expands Th1 immunity upon subsequent infection

**Fig 1:**
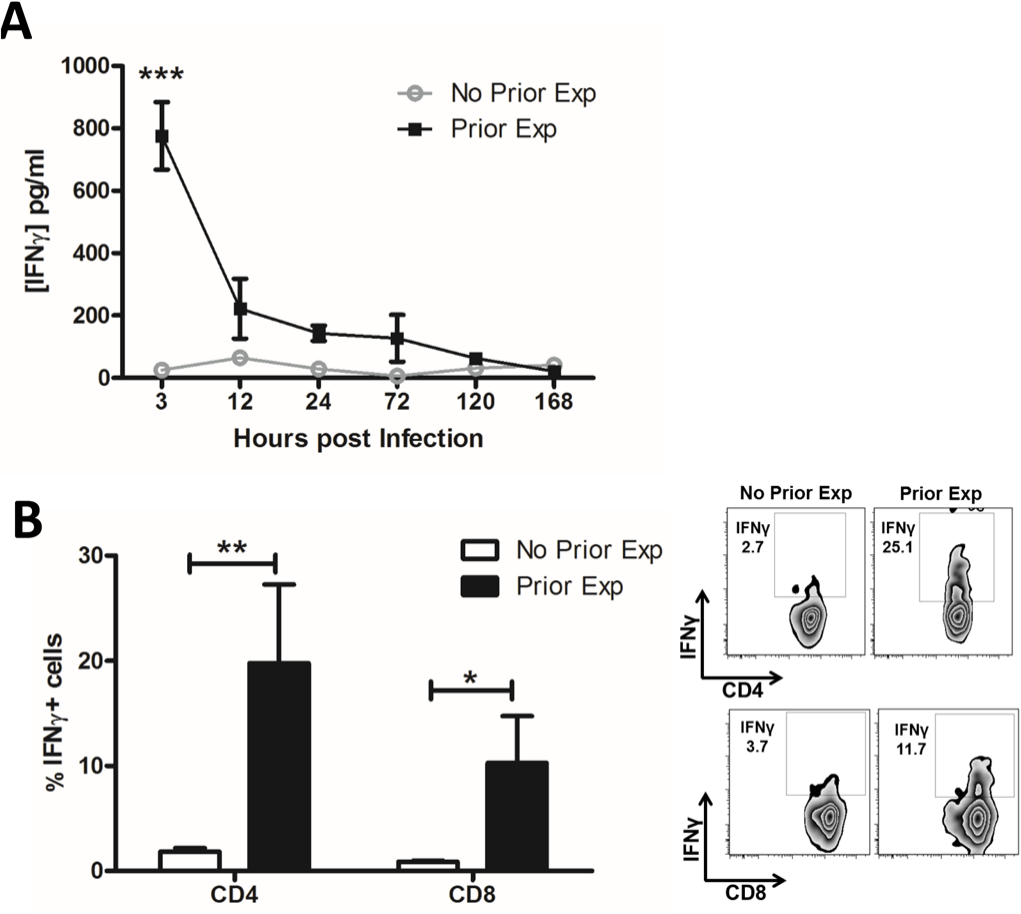
Prior exposure to *S*. *aureus* increases IFNγ secretion by CD4^+^ and CD8^+^ T cells during subsequent infection. Groups of mice were exposed to *S*. *aureus* (5x10^8^ CFU) via an i.p. injection on d 0, 7 and 14. Prior exposed mice were then re-challenged with an i.p. injection of *S*. *aureus* (5x10^8^ CFU) on d 35 alongside a control group of naïve mice. At indicated time points post-challenge the peritoneal cavity was lavaged with PBS to assess IFNγ secretion by ELISA (A). n = 15 per group. At 3 h post challenge peritoneal cells were isolated to assess the proportions of IFNγ-producing CD4^+^ and CD8^+^ T cells using flow cytometry (B). Results expressed as mean ± SEM and representative FACS plots. n = 5 per group. *p<0.05, **p<0.005, ***p<0.001.

Using a previously described [30] murine model of recurrent *S*. *aureus* exposure, followed by a period of recovery prior to subsequent challenge, we identified that local production of IFNγ in the peritoneal cavity was significantly increased soon after challenge of prior exposed mice, while it remained undetectable in naïve mice infected for the first time (Fig 1A). Twenty percent of CD4^+^ and 10% of CD8^+^ T cells in the peritoneal cavity were making IFNγ (Fig 1B). We have previously shown that γδ^+^ T cells were not contributing to IFNγ production in this model [30]. Similarly, there was no evidence for IFNγ production by NK cells. Thus, CD4^+^ T cells were the primary source of IFNγ in the prior exposed mice. CD4^+^ T cells–but not CD8^+^ T cells–with the capacity for IFNγ production were sustained in the peritoneum for up to 5 d post-challenge (S1A Fig). CD4^+^IFNγ^+^ (Th1) cells were evident in the spleen by 12 h post-challenge, with no similar systemic expansion of CD8^+^ cells (S1B Fig). Neither CD4^+^ nor CD8^+^ IFNγ-producing cells were found in the draining mediastinal lymph nodes (MLN).

With the exception of TNFα, innate cytokine secretion (IL-1β, IL-23, and IL-12) in the peritoneal cavity did not differ between naive and prior exposed mice at 3 h post-challenge (S1C Fig). IL-1α and IL-18 were also undetectable in both groups of mice. This suggests that the enhanced T cell production of IFNγ observed in prior exposed mice was not directly related to increased innate cytokine signalling, and indeed these Th1 cells may contribute to the elevated levels of TNFα observed. Repeated exposure to *S*. *aureus* preferentially enhanced local and systemic Th1 (CD4^+^IFNγ^+^) responses, which likely contributed to the accelerated bacterial clearance seen during subsequent *S*. *aureus* infection [30]. We have previously shown that IL-17 levels are also significantly elevated in previously exposed mice and that γδ^+^ T cells, as opposed to CD4^+^ T cells, are the primary source [30]. The Th2 cytokine IL-4 was undetectable in both groups of mice.

### Adoptive transfer of *S*. *aureus* antigen-specific Th1 cells accelerates clearance of infection by enhancing the macrophage response

**Fig 2:**
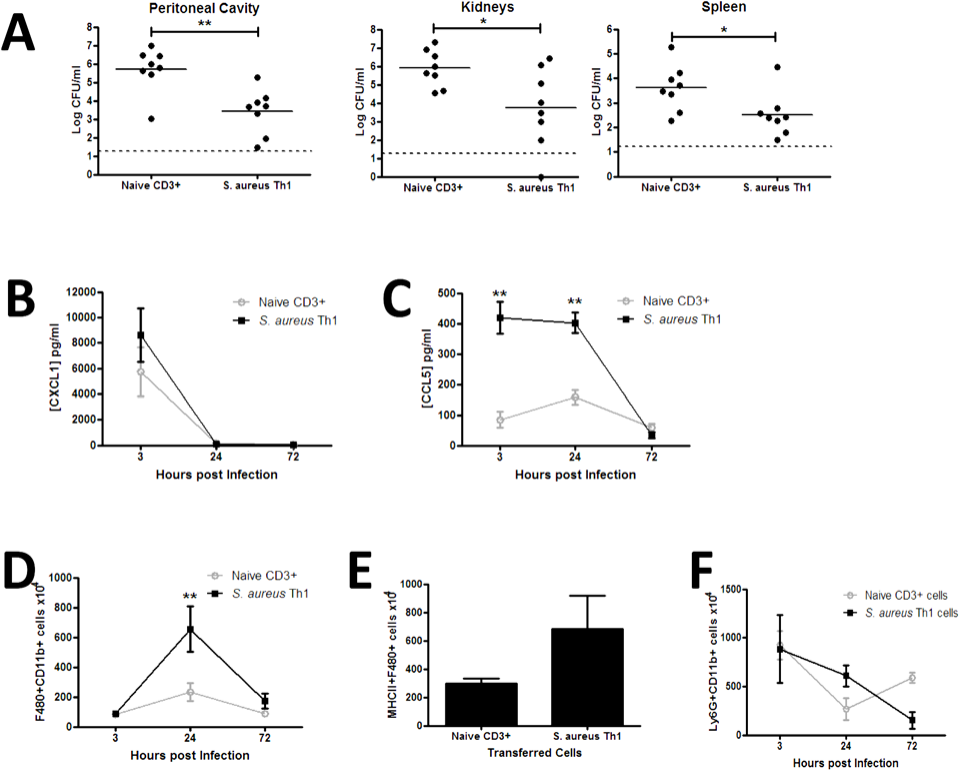
Transfer of *S*. *aureus* antigen-specific peritoneal Th1 cells protects against subsequent *S*. *aureus* infection via enhanced macrophage responses. Groups of mice received transfers of 5x10^6^ *S*. *aureus* specific Th1 cells originating from the peritoneal cavity of previously exposed mice via i.p. injection. Another group of mice received a transfer of 5x10^6^ naive splenic CD3^+^ cells as a control. At 3 h post transfer both groups of mice were challenged with *S*. *aureus* (5x10^8^ CFU) via i.p. injection. At 72 h post-bacterial challenge the bacterial burden was assessed in the peritoneal cavity, kidneys and spleen (A). Results expressed as log_10_ CFU/ml with mean indicated by bars. At indicated time points post-bacterial challenge, the peritoneal cavity was lavaged with PBS to assess CXCL1 and CCL5 secretion by ELISA (B,C). Results expressed as mean ± SEM. At indicated time points post-challenge, the absolute numbers of macrophages (F4/80^+^Ly6G^-^) were assessed in the peritoneal cavity by flow cytometry (D). MHCII expression by infiltrating macrophages was determined 24 h post infection (E). Absolute numbers of neutrophils (Ly6G^+^CD11b^+^) in the peritoneal cavity were assessed at the indicated time points post-challenge (F). Results expressed as mean ± SEM. n = 5–8 mice per group. Data pooled from 3 independent experiments. *p<0.05, **p<0.005.

To establish whether *S*. *aureus* antigen-specific Th1 cells are capable of conferring protection against infection, we expanded *S*. *aureus* antigen-specific T cells under Th1-polarising conditions *in vitro* and then adoptively transferred these cells to naïve mice. Prior to transfer it was confirmed that these Th1-polarised cells were producing significant levels of IFNγ and only background levels of other cytokines (S2A Fig), and greater than 99% of the CD4^+^ T cells transferred were viable. *S*. *aureus* antigen-specific Th1 cells, or an equivalent number of naïve non-polarised non-antigen-specific T cells, were then adoptively transferred to naïve syngeneic hosts prior to intraperitoneal (i.p.) *S*. *aureus* challenge. At 24 h, there was no difference in bacterial burden in the peritoneal cavity (5.7 log_10_ CFU/ml versus 6.1 log_10_ CFU/ml) or kidney (4.4 log_10_ CFU/ml versus 4.8 log_10_ CFU/ml) between *S*. *aureus* Th1 cell transfer and control cell transfer groups, respectively. However, *S*. *aureus*-specific Th1 cell transfer significantly reduced bacterial burden (~2 log_10_ reduction) in the peritoneal cavity at 72 h post-challenge as compared with transfer of control T cells. Dissemination of bacteria to the kidneys and spleen was also reduced in recipients of *S*. *aureus* antigen-specific Th1 cells at 72 h post-challenge (Fig 2A). OVA-specific Th1 cells were polarised under similar conditions, and produced significant levels of IFNγ (41 ng/ml), but minimal IL-17, *in vitro*. Importantly, transfer of these OVA-specific Th1 cells did not confer protection against systemic dissemination of *S*. *aureus*, illustrating the specificity of the response (S2B Fig).

To establish a mechanism for the Th1-conferred protective effect, we assessed phagocyte responses at the site of infection. Transfer of *S*. *aureus* antigen-specific Th1 cells did not affect local production of the neutrophil-recruiting chemokine CXCL1 (Fig 2B), but was associated with a significant elevation in CCL5 secretion in the peritoneal cavity early in the course of infection (Fig 2C), inferring a bias towards monocyte and memory T cell recruitment. The effect of local chemokine production on downstream phagocyte influx was then established. Macrophage (CD11b^+^F480^+^Ly6G^-^) recruitment to the site of infection was significantly elevated in Th1 transfer recipients by 24 h post-infection (Fig 2D), with the number of MHC II-expressing macrophages also increased (Fig 2E). Consistent with the CXCL1 result, neutrophil (CD11b^+^F480^-^Ly6G^+^) recruitment to the peritoneal cavity was not significantly altered in the Th1 transfer recipients as compared to the control mice (Fig 2F). Transfer of OVA-specific Th1 cells did not result in increased activation of recruited macrophages, as evidenced by comparable MHC II expression to PBS-treated controls (S2C Fig).

To confirm that the protective effects observed in the Th1 recipient mice were exclusively mediated by IFNγ-producing cells, we demonstrated that local IL-17 production was not increased in the Th1 recipient mice as compared to control mice (S2D Fig). Furthermore, transfer of *S*. *aureus* antigen-specific Th17 cells (S3A Fig) did not reduce bacterial burden at any site in the recipient mice (S3B Fig). Taken together, these results suggest that the presence of *S*. *aureus* antigen-specific Th1 cells promotes monocyte/macrophage activation, facilitating accelerated clearance of infection at local and systemic sites.

### Macrophages are critically important in protection against *S*. *aureus* infection

**Fig 3:**
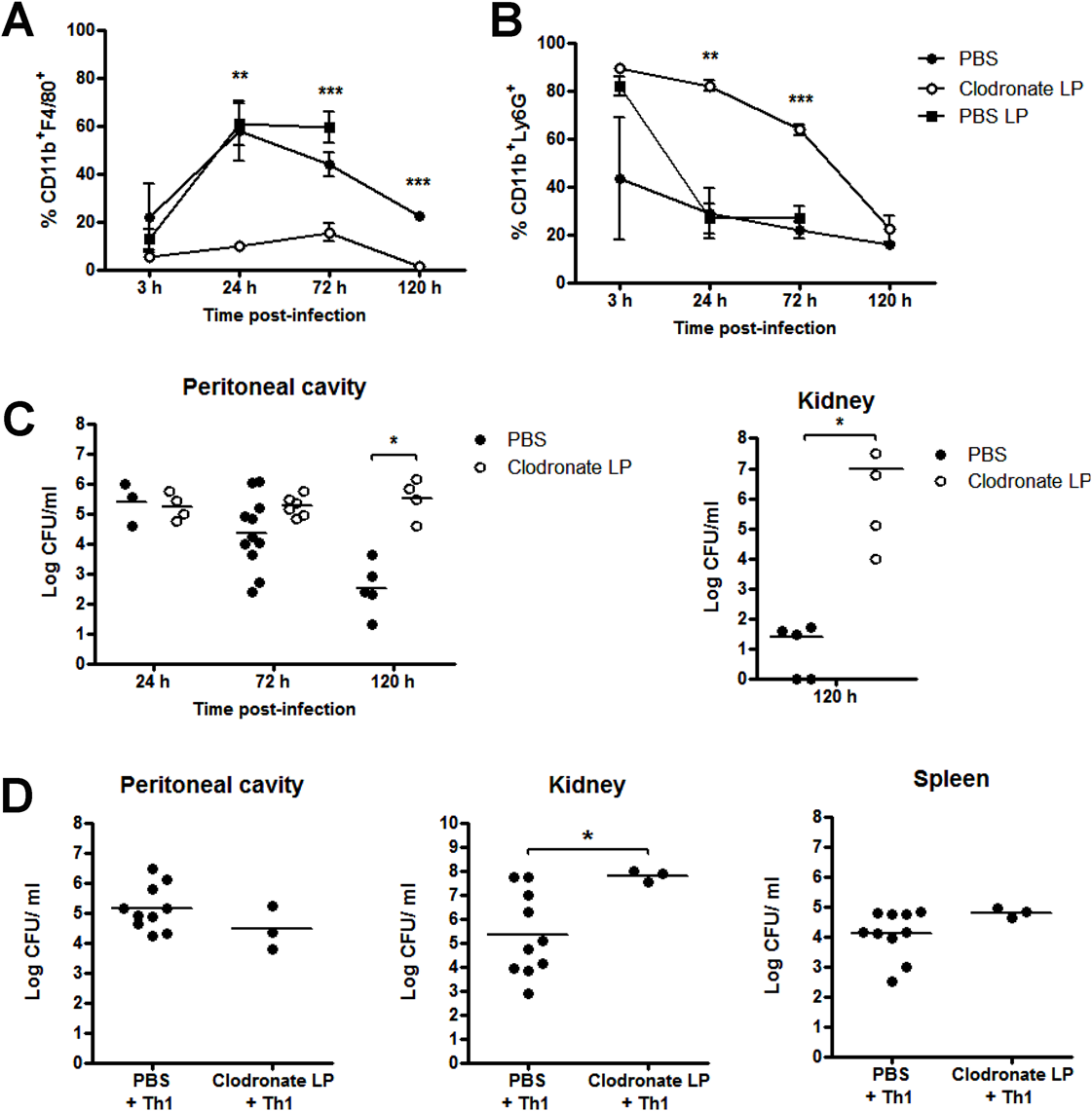
Macrophages are critically important in protection against *S*. *aureus* infection. Groups of mice were administered 200 μl clodronate liposomes (LP), PBS LP or PBS i.p. at 0 h and 72 h. 24 h after initial clodronate treatment, all mice were challenged with *S*. *aureus* (5x10^8^ CFU) via i.p. injection. At the indicated time-points post-bacterial challenge, the peritoneal cavity was lavaged with PBS and the frequency of macrophages (CD11b^+^F4/80^+^Ly6G^-^) (A) and neutrophils (CD11b^+^F4/80^-^Ly6G^+^) (B) were assessed by flow cytometry. Results expressed as mean ± SEM. Bacterial burden in the peritoneal cavity and kidneys was also assessed at the indicated time points post-bacterial challenge (C). Results expressed as log_10_ CFU/ml with mean indicated by bars. Data pooled from 3 independent experiments. n = 4–11 mice per group. Further groups of mice were administered 200 μl clodronate LP or PBS i.p., and 24 h later, both groups received *S*. *aureus*-specific Th1 cells (5x10^6^) i.p., originating from the peritoneal cavity of previously exposed mice. 3 h post-Th1 transfer, all mice were challenged with *S*. *aureus* (5x10^8^ CFU) via i.p. injection. At 72 h post-challenge, bacterial burden in the peritoneal cavity, kidneys and spleen was assessed (D). Results expressed as log_10_ CFU/ml with mean indicated by bars. Data pooled from 2 independent experiments, n = 3–10 mice per group. *p<0.05, **p<0.005, ***p<0.001.

To further investigate the role of macrophages in the immune response against *S*. *aureus*, we depleted peritoneal macrophages using clodronate-loaded liposomes prior to infection. This treatment completely depleted macrophages in the peritoneum by 24 h post-administration, compared to control mice injected with PBS-loaded liposomes or PBS alone (Fig 3A). Following *S*. *aureus* infection, macrophages were rapidly recruited to the peritoneum in control mice, whereas no macrophage recruitment was observed in clodronate-treated mice. Conversely, significant peritoneal neutrophil recruitment was observed in the first 24 h post-treatment with either liposomal clodronate or liposomal PBS (Fig 3B). While neutrophil numbers in liposomal PBS-treated mice had returned to PBS control levels 24 h post-*S*. *aureus* challenge, neutrophils in clodronate-treated mice remained significantly higher than control mice until 72 h post-infection (Fig 3B). Despite this elevated neutrophil response, the macrophage-depleted animals demonstrated a significant impairment in their ability to control *S*. *aureus* infection. By day 5 post-challenge, bacterial burdens in the peritoneal cavity and in the kidneys were significantly elevated as compared to PBS-treated controls (Fig 3C). In addition, 26% of macrophage-depleted mice subsequently infected with *S*. *aureus* had died by 72 h post-challenge. There were no deaths in the liposomal PBS or PBS-treated cohorts.

Having shown that macrophage responses were crucial to controlling *S*. *aureus* infection, we next looked to confirm that *S*. *aureus-*specific Th1 cells were positively associated with this macrophage recruitment or activation. We adoptively transferred *S*. *aureus-*specific Th1 cells to groups of macrophage-depleted (clodronate-treated) mice, before *S*. *aureus* challenge. In the presence of clodronate liposomes, Th1 cells failed to control systemic bacterial infection. Fifty percent of the mice had died and there were significantly elevated levels of bacteria present in the kidneys (of surviving mice) at 72 h post-bacterial challenge, compared to PBS-treated controls (Fig 3D). Together, these results highlight the critical role of macrophages in containing *S*. *aureus* infection and suggest that macrophages play an active role in direct bacterial clearance. We have recently demonstrated that macrophages possess a significant capacity for direct killing of *S*. *aureus* [35], and our preliminary experiments have shown that treatment of murine peritoneal macrophages with recombinant IFNγ enhanced their capacity to kill *S*. *aureus in vitro* [64.6% vs 81.37% killing of *S*. *aureus* strain PS80 (multiplicity of infection 1).

### Human *S*. *aureus* bloodstream infection induces *S*. *aureus* antigen-specific memory Th1 cells

Human adaptive cellular immune responses to *S*. *aureus* infection have not been previously examined. To investigate whether our murine findings translated to humans, thirty-five immunocompetent adult patients with bloodstream infection were recruited between February 2013 and November 2014 from three tertiary care centres. Twenty-four *S*. *aureus* (SA) and eleven *Escherichia coli* (EC) BSI patients who met inclusion criteria (S1 Table) consented to participate. Patient demographics are listed in Table 1.

**Table 1:** Patient demographics. Data are displayed as median (interquartile range) and number (percentage). *P* values are calculated by Mann-Whitney and Fisher’s exact test respectively.

	*S*. *aureus* (n = 24)	*E*. *coli* (n = 11)	p-value
Age	48 (31–71)	75 (57–83)	0.06
Male	17 (71%)	3 (27%)	0.03
MRSA	4 (17%)	NA	
**Co-morbidities**			
Haemodialysis	4 (17%)	0 (0%)	0.28
Diabetes mellitus	6 (25%)	1 (9%)	0.39
**Acquisition** ^**a**^			
Healthcare-associated	12 (50%)	2 (18%)	0.14
**Source**			
Unknown	7 (29%)	1 (9%)	0.39
Central venous catheter	6 (25%)	0 (0%)	0.15
Peripheral venous catheter	3 (12.5%)	0 (0%)	0.54
Injection drug use	5 (21%)	0 (0%)	0.16
Diabetic foot ulcer ^†^	3 (12.5%)	0 (0%)	0.54
Urinary tract	0 (0%)	9 (82%)	<0.001
Biliary tract	0 (0%)	1 (9%)	0.31
**Complicated *S*. *aureus* BSI** ^**b**^	**19 (79%)**	NA	
Infective Endocarditis	4 (17%)		
Septic Thrombophlebitis	5 (21%)		
Haematogenous osteo-articular infection	7 (29%)		
Contiguous osteomyelitis^†^	3 (12.5%)		

^a^ Healthcare-associated infections were defined as (i) index positive blood culture collected ≥48hrs after hospital admission, and no signs or symptoms of the infection noted at time of admission; OR (ii) index positive blood culture collected <48hrs after hospital admission if any of the following criteria are met: received intravenous therapy in an ambulatory setting in the 30 days before onset of BSI, attended a hospital clinic or haemodialysis in the 30 days before onset of BSI, hospitalised in an acute care hospital for ≥ 2 days in the 90 days prior to onset of BSI, resident of nursing home or long-term care facility.

^b^ *Staphylococcus aureus* bacteraemia was defined as uncomplicated if all of the following criteria were met: exclusion of endocarditis; no evidence of metastatic infection; absence of implanted prostheses; follow-up blood cultures at 2–4 days culture-negative for *S*. *aureus*; defervescence within 72 h of initiating effective therapy. Percentages shown are of entire *S*. *aureus* BSI population.

^†^ Three patients had chronic diabetic foot ulcers as a source of their *S*. *aureus* BSI, and in all cases the contiguous underlying bone was also found to be infected.

MRSA = methicillin-resistant *Staphylococcus aureus*. NA = not applicable. BSI = bloodstream infection.

**Fig 4:**
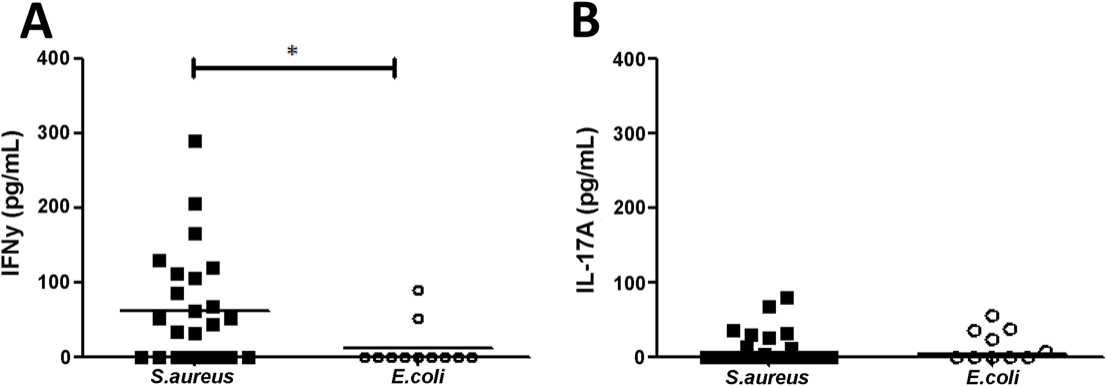
Human *S*. *aureus* bloodstream infection is associated with increased IFNγ production. Serum from *S*. *aureus* and *E*.*coli* bloodstream infection patients was collected on day 7 ± 2 post-initial bacteraemia and assessed for IFNγ (A) and IL-17A (B) by ELISA. Results expressed as individual patient values with median indicated by bar, n = 11–24 per group. *p<0.05.

Initial cytokine analysis of serum from BSI patients demonstrated that IFNγ was detectable in 60% (n = 15) of SA BSI and only 18% of EC BSI patients (p = 0.03). SA BSI patient serum showed significantly higher levels of IFNγ than EC patients (Fig 4A). Low levels of IL-17A were detectable in the sera of a minority of patients, but these levels did not differ significantly between groups (Fig 4B).

Having observed that signature T cell cytokines were present and elevated in some SA BSI patients, we proceeded to establish if antigen-specific T helper cell populations were expanded. This strategy may be more sensitive than serum ELISA at detecting differences as it can discriminate cytokine production at a single-cell level. Human T cell proliferative and cytokine responses are known to be significantly reduced following sepsis [36], and as such healthy volunteers may be a suboptimal control group for comparing antigen-specific responses in infected patients. To confirm this, we measured proliferative responses to a superantigen (staphylococcal enterotoxin A, SEA) in a subgroup of patients and healthy volunteers. Non-specific CD4^+^ T cell proliferation was significantly reduced in SA and EC BSI patients compared to healthy volunteers (S4A Fig). Consequently, the *E*. *coli* BSI cohort was chosen as the more appropriate control group to establish antigen-specific T cell responses during BSI, despite some expected clinical differences between the groups.

Whole-genome sequencing of *S*. *aureus* bloodstream isolates showed that clonal complex (CC) 5 accounted for the biggest group of isolates (31.8%, n = 7), followed by CC22 (22.8%, n = 5) and CC1 (13.6%, n = 3). The majority (83%, n = 20) of BSI strains were methicillin-sensitive (MSSA). All cases of methicillin-resistant SA BSI were healthcare-associated and belonged to CC22. The clinical *S*. *aureus* bloodstream isolates displayed notable genetic diversity, and also differed from the two laboratory reference strains (S5 Fig). *S*. *aureus* antigen-specific CD4^+^ T cell responses were determined by incubating CFSE-labelled PBMCs with each patient’s own infecting strain and a panel of reference strains of heat-killed *S*. *aureus*, resulting in a challenge of four or five different CC types per patient. Despite this diverse challenge, intra-individual patient responses to the various *S*. *aureus* strains did not differ significantly (S2 Table), reflecting the fact that most potential epitopes are likely conserved across strains. Therefore results are presented as pooled responses to all strains of heat-killed *S*. *aureus*.

**Fig 5:**
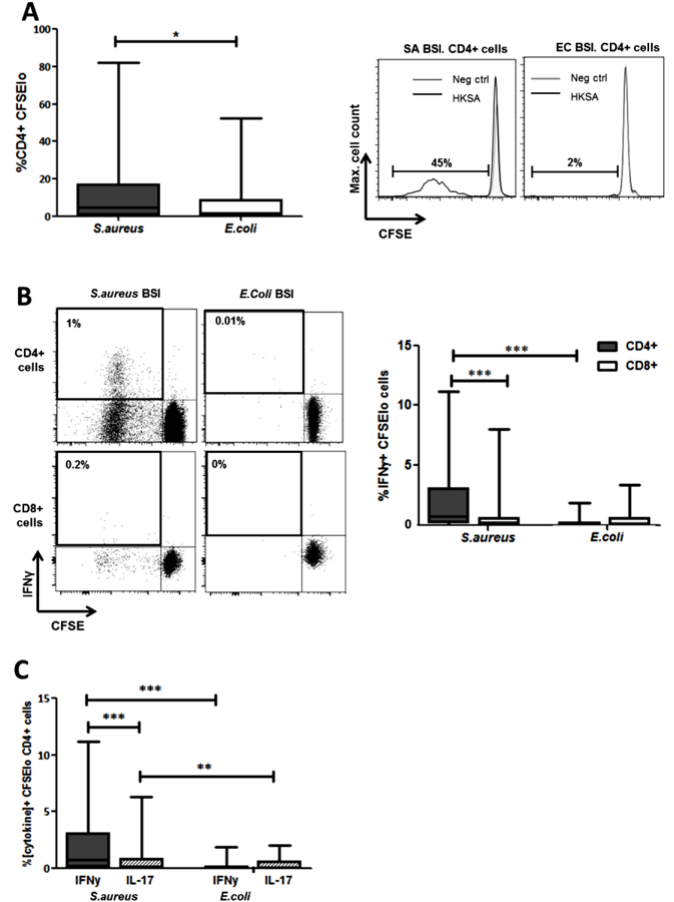
Human *S*. *aureus* bloodstream infection induces *S*. *aureus* antigen-specific Th1 cells. PBMCs were isolated from patients, CFSE-labelled and incubated with heat-killed *S*. *aureus* PS80 strain (1μg/ml ≈ 1x10^7^ CFU/ml) or media alone for 10 d before assessing *S*. *aureus* antigen-specific proliferation by gating on CFSE_lo_ cells of the CD4^+^ population using flow cytometry (A). *S*. *aureus* antigen-specific Th1 and cytotoxic T cell division was assessed by gating on CFSE_lo_ IFNγ^+^ cells of the CD4^+^ and CD8^+^ populations respectively (B). *S*. *aureus* antigen-specific Th1 and Th17 proportions were compared by gating on CFSE_lo_ IFNγ^+^ or IL-17A^+^ cells in the CD4^+^ population (C). For each patient, media only responses were subtracted from responses to heat-killed *S*. *aureus* to determine the antigen-specific response. Results shown as box-and-whiskers plots where the horizontal line indicates the median, boundaries of the box indicate the IQR, and whiskers indicate the highest and lowest values of the results, and representative FACS plots of CD4^+^ or CD8^+^ lymphocytes (A, B). n = 5–17 per group. SA = *S*. *aureus*, EC = *E*. *coli*, BSI = bloodstream infection. *p<0.05, **p<0.005, ***p<0.001.

The proportion of CD4^+^ T cells proliferating in response to *in vitro S*. *aureus* re-stimulation was significantly elevated in the SA BSI patients compared to the EC BSI patients (Fig 5A). In addition, the proportion of CD4^+^ T cells showing both proliferation and secretion of IFNγ was significantly greater among SA patients, and *S*. *aureus* antigen-specific Th1 responses were almost completely lacking in EC BSI patients (Fig 5B). Interestingly, in a subset of patients approximately 14% of the proliferating Th1 (IFNγ^+^CD4^+^) cells were co-producing TNFα, suggesting that polyfunctional T cells may be involved in the immune response to *S*. *aureus* infection. *S*. *aureus* antigen-specific CD8^+^IFNγ^+^ T cells were minimal in both patient groups (Fig 5B). CD4^+^ T cell proliferation in response to *in vitro* stimulation with heat-killed *E*.*coli* was minimal and did not differ significantly between the two patient groups (S4B Fig).

Despite the fact that serum IL-17 levels in both groups were low or undetectable, we investigated for the presence of *S*. *aureus* antigen-specific Th17 cells. Th17 cell expansion following *S*. *aureus* re-stimulation was significantly lower than the predominant Th1 expansion already described. However, these *S*. *aureus* antigen-specific Th17 cells were preferentially expanded in SA BSI patients and minimally present in EC BSI patients (Fig 5C).

**Fig 6:**
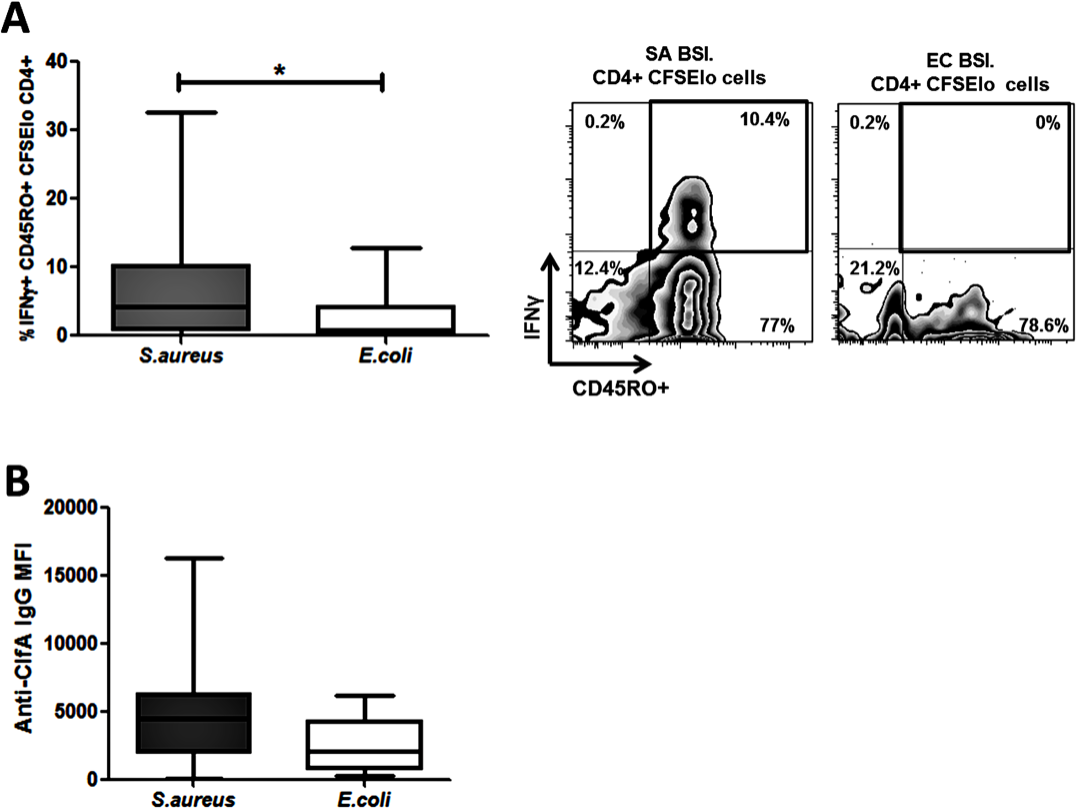
Human *S*. *aureus* bloodstream infection induces *S*. *aureus* antigen-specific effector memory Th1 cells and anti-ClfA antibodies. PBMCs were isolated from patients, CFSE-labelled and incubated with heat-killed *S*. *aureus* PS80 strain (1μg/ml ≈ 1x10^7^ CFU/ml) or media alone for 10 d. Proportions of *S*. *aureus* antigen-specific effector memory cells were assessed by gating on IFNγ^+^CD45RO^+^ cells in the CFSE_lo_ CD4^+^ population (A). For each patient, media only responses were subtracted from responses to heat-killed *S*. *aureus* to determine the antigen-specific response. n = 5–12 per group. IgG antibody binding to ClfA was measured in patient sera using a bead-based flow cytometry technique (B). n = 11–24 per group. Results shown as box-and-whiskers plots where the horizontal line indicates the median, boundaries of the box the IQR, and whiskers indicate the highest and lowest values of the results, and representative FACS plots of CD4^+^CFSE_lo_ cells (A). SA = *S*. *aureus*, EC = *E*. *coli*, BSI = bloodstream infection. * p<0.05.

To establish if proliferating cells possessed a memory phenotype we investigated expression of CD45RO–a marker of previously activated or antigen-experienced T cells. The majority of the proliferating antigen-specific CD4^+^ T cells present were CD45RO^+^. In SA BSI patients, the proportion of these cells that produced IFNγ was significantly elevated as compared to the EC BSI patients (Fig 6A).

Taken together, these results demonstrate for the first time that invasive *S*. *aureus* infection in humans predominantly expands a population of memory Th1 cells (with Th17 cells expanded to a lesser extent). These cells are primed and expanded *in vivo* in patients with recent bloodstream infection and thus may play a role in recovery.

### Staphylococcal Clumping factor A drives antigen-specific Th1 responses in human CD4^+^ T cells

The staphylococcal cell wall-anchored protein clumping factor A (ClfA) has been proposed as a promising target for inclusion in multivalent vaccines given its importance as a virulence factor and its ubiquitous expression among clinical isolates [37]. Whole-genome sequencing revealed the presence of the *clfA* gene in all *S*. *aureus* bloodstream isolates in our patient cohort. We then confirmed that ClfA was expressed by the four reference *S*. *aureus* strains used in our patient assays (S6A Fig), and remained present on the bacterial cell surface after heat-killing (S6B Fig). This indicated that the antigen-specific T cell response seen in patients could be at least partially attributable to ClfA. In our SA BSI patient cohort, serum anti-ClfA antibodies were elevated more than in the EC BSI group, validating its humoral immunogenicity (Fig 6B).

**Fig 7:**
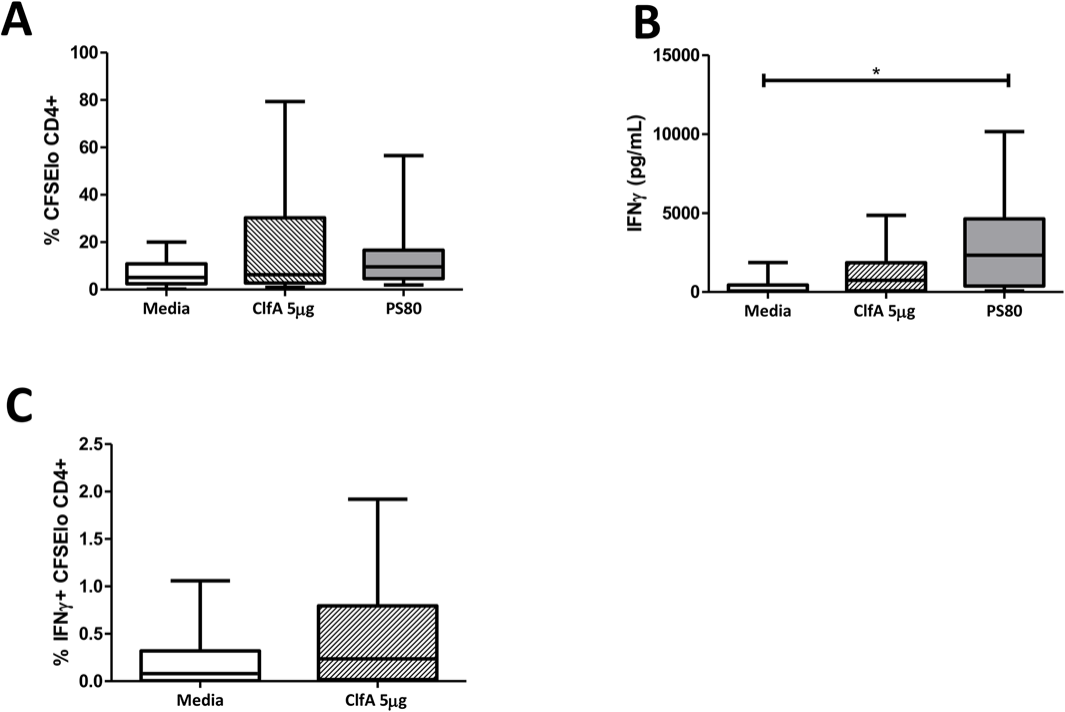
Human CD4^+^ T cells show ClfA-specific type 1 immune responses. Purified CD4^+^ T cells and irradiated APCs from healthy blood donor buffy coats were CFSE-labelled and co-cultured with ClfA (5μg/ml), heat-killed *S*. *aureus* PS80 strain (1μg/ml ≈ 1x10^7^ CFU/ml), or media alone. After 10 d, proliferation was assessed by gating on CFSE_lo_ cells in the CD4^+^ population (A). At 96 h cell supernatants were collected to assess IFNγ secretion by ELISA (B). ClfA-specific Th1 cell division was assessed at d 10 by gating on CFSE_lo_ IFNγ^+^ cells in the CD4^+^ population (C). Results expressed as mean ± SEM. n = 9–14 per group.

To confirm that ClfA had capacity for human T cell activation, CD4^+^ T cells were isolated from buffy coats of healthy adult blood donors and co-cultured with autologous irradiated APCs in the presence or absence of antigen. Given that the normal healthy population have multiple prior exposures to *S*. *aureus*, immune recall responses to staphylococcal antigens are expected but are likely to vary significantly, depending on individuals’ exposure histories. Indeed, humoral responses (in healthy volunteers and patients with *S*. *aureus* BSI) [9] and cellular responses (in healthy volunteers only) [34] have been demonstrated to a limited number of antigens. Thus, the use of buffy coats presents a valuable opportunity to screen antigens for T cell-activating capacity. Stimulation with purified ClfA induced antigen-specific CD4^+^ T cell expansion of a similar order to that of heat-killed *S*. *aureus* in 71% of individuals, while 88% of individuals were in possession of T cells capable of responding to heat-killed *S*. *aureus* (Fig 7A). Substantial IFNγ secretion was also seen in response to stimulation by ClfA and heat-killed *S*. *aureus* (Fig 7B), and we confirmed that a proportion of CD4^+^ T cells were both proliferating and producing IFNγ in response to ClfA (Fig 7C). In this assay system, ClfA-specific Th17 cells were not evident by flow cytometry and IL-17 was not detectable in the culture supernatant by ELISA.

### Immunisation with a novel anti-*S*. *aureus* vaccine comprising *S*. *aureus* surface protein ClfA and TLR9 agonist CpG promotes Th1 immune responses

Having confirmed the human T cell-activating potential of the *S*. *aureus* surface antigen ClfA, we designed a model *S*. *aureus* vaccine comprising ClfA formulated with CpG as an adjuvant to induce Th1 responses. Groups of naïve mice were vaccinated with CpG alone, CpG+ClfA, or PBS alone. ClfA-specific humoral and T cell responses were assessed post-vaccination. ClfA+CpG drove a marked increase in IFNγ production by inguinal lymph node (ILN) cells following 72 h *in vitro* restimulation with ClfA (10μg/ml) (51 vs. 16 pg/ml, for ClfA+CpG vs. CpG-vaccinated mice respectively) and spleen cells (219 vs. 2 pg/ml for ClfA+CpG vs. CpG-vaccinated mice), with an IL-17 response in ILN cells only (84 vs. 22 pg/ml for ClfA+CpG vs. CpG-vaccinated mice).

**Fig 8:**
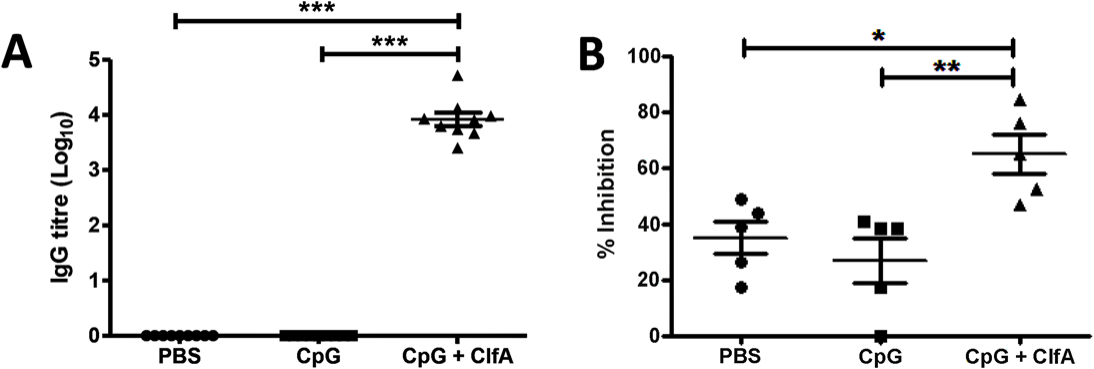
Vaccination with CpG and ClfA induces ClfA-specific cellular and humoral responses. Mice were vaccinated with CpG (50μg/mouse), CpG+ClfA (50μg+1μg/mouse) or PBS via s.c. injection on d 0, 14 and 28. On d 63 sera was collected from vaccinated mice. ClfA-specific IgG titres were determined using ELISA (A). Neutralising antibodies were determined by measuring the ability of serum to inhibit staphylococcal adherence to fibrinogen via ClfA (B). Results expressed as mean ± SEM. n = 5 per group. *p<0.05, **p<0.005, ***p<0.001.

Anti-ClfA IgG titres were significantly elevated in the sera of ClfA+CpG-immunised mice as compared to sham-immunised (PBS alone or CpG alone) mice (Fig 8A). Neutralising antibodies were also present in the sera of immunised mice and effectively inhibited the binding of ClfA to fibrinogen (Fig 8B).

### A Th1-inducing vaccine accelerates bacterial clearance in mice during systemic *S*. *aureus* infection via enhanced macrophage responses

**Fig 9:**
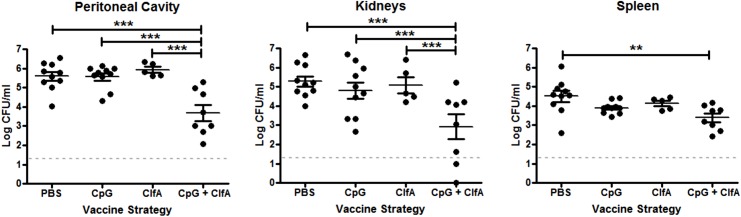
Vaccine-induced type 1 immunity protects against *S*. *aureus* infection. Mice were vaccinated with CpG (50μg/mouse), ClfA (1μg/mouse), or CpG+ClfA via s.c. injection on d 0, 14 and 28. On d 63 mice were challenged with *S*. *aureus* (5x10^8^ CFU) via i.p. injection alongside a control group of sham-immunised (with PBS) mice. At 72 h post-infection, bacterial burden was assessed in the peritoneal cavity, kidneys and spleen. Results expressed as log_10_ CFU/ml with means indicated by bars. n = 5–10 per group. **p<0.005, ***p<0.001.

Having confirmed that immunisation with ClfA+CpG successfully induced antigen-specific Th1 cellular and humoral immunity, we sought to establish if such vaccine-induced immunity could protect against *S*. *aureus* challenge. The efficacy of this vaccine against systemic *S*. *aureus* infection was shown by the significant reduction in bacterial burden at 72 h post challenge at the peritoneal infection site (~2 log_10_), as well as systemically in the kidneys (~2.5 log_10_) and spleen (~1 log_10_) in mice that were immunised with ClfA+CpG as compared to those that received antigen alone, adjuvant alone or vehicle (Fig 9). No protection was seen at 24 h post-challenge in the peritoneal cavity (4.5 log_10_ CFU/ml versus 5.2 log_10_ CFU/ml) or kidney (3.5 log_10_ CFU/ml versus 3.7 log_10_ CFU/ml) in ClfA+CpG immunised as compared to control mice, respectively.

**Fig 10:**
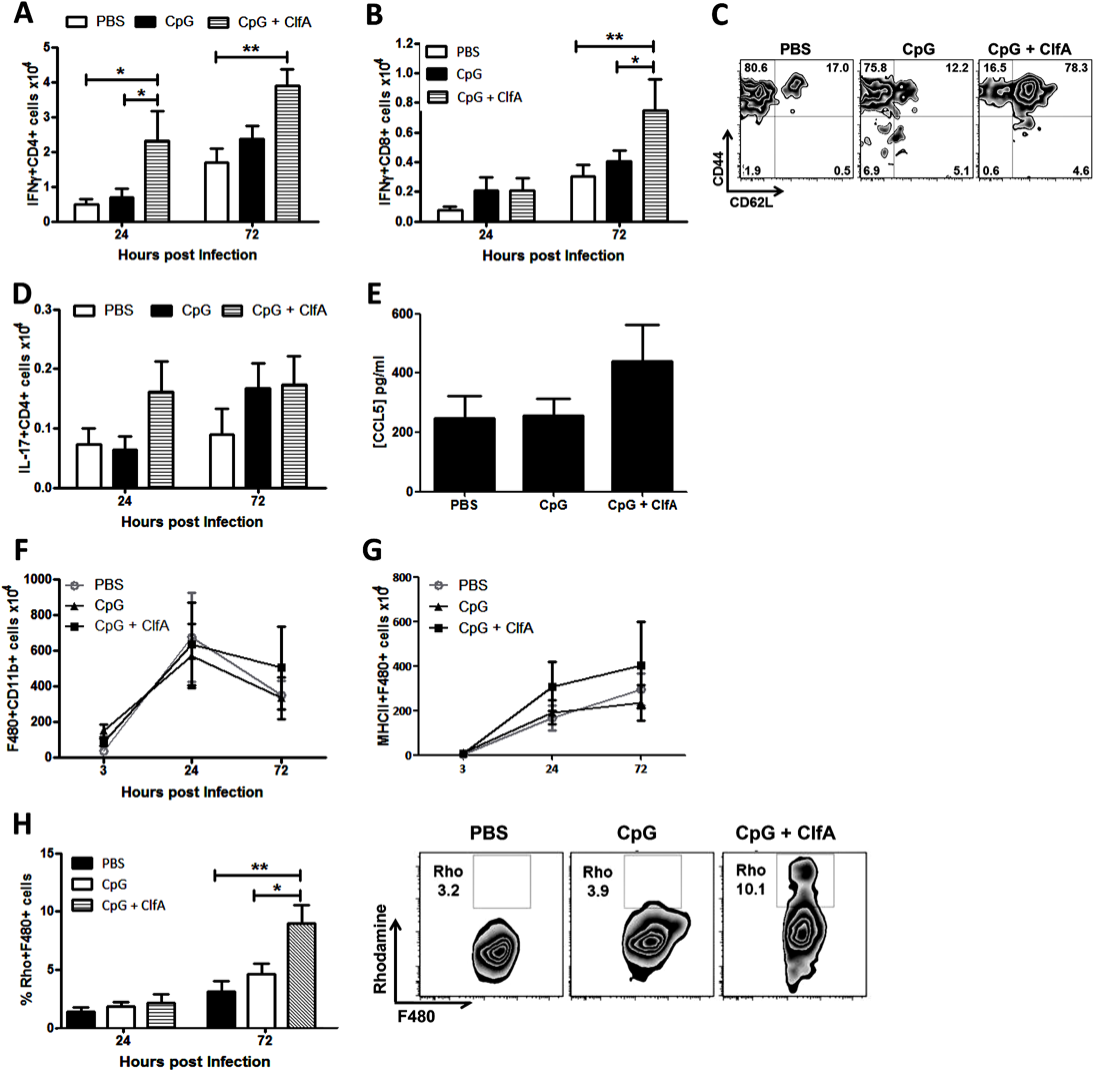
Vaccination with CpG and ClfA induces ClfA-specific type 1 immunity. Mice were vaccinated with CpG (50μg/mouse) or CpG+ClfA (50μg+1μg/mouse) via s.c. injection on d 0, 14 and 28. On d 63 mice were challenged with *S*. *aureus* (5x10^8^ CFU) via i.p. injection. At 24 h and 72 h post-infection cells were isolated from the peritoneal cavity to assess absolute numbers of IFNγ^+^CD4^+^ (A) and IFNγ^+^CD8^+^ (B) cells. At 72 h post-infection the proportions of IFNγ^+^CD4^+^ cells in the peritoneum expressing the memory markers CD44 and CD62L was assessed by flow cytometry (C). Numbers of IL-17^+^CD4^+^ T cells in the peritoneum were also assessed at the indicated time points (D). At 24 h post-challenge the peritoneal cavity was lavaged with PBS to assess CCL5 secretion by ELISA (E). At indicated time points post-challenge, the absolute numbers of macrophages (F4/80^+^CD11b^+^) were assessed in the peritoneal cavity by flow cytometry (F). Numbers of infiltrating MHCII^+^ macrophages were also determined at indicated time points post-infection (G). Results expressed as mean ± SEM and representative FACS plots 72 h post-challenge. Data pooled from 2 independent experiments, n = 5–10 per group. At indicated time points post-challenge, macrophages isolated from the peritoneal cavity were assessed for reactive oxygen species (ROS) activity, detected by rhodamine (Rho) positive cells (H). Results expressed as mean ± SEM and representative FACS plots 72 h post-challenge. n = 5 per group. *p<0.05, **p<0.005.

To determine if this vaccine-induced protection was associated with an increased Th1 response, the cells infiltrating the peritoneal cavity post-infection were assessed. CD4^+^IFNγ^+^ cells were significantly increased in the peritoneal cavities of ClfA+CpG-immunised mice compared to control groups at both 24 and 72 h post-challenge (Fig 10A). CD8^+^IFNγ^+^ cells were increased in these mice by 72 h (Fig 10B). The memory phenotype of these responding Th1 cells was confirmed by their expression of CD44 and CD62L –effector memory cells being identified as CD44^hi^CD62L^lo^ and central memory cells as CD44^hi^CD62L^hi^. Thus, ClfA+CpG vaccine recipients showed a marked Th1 total memory (CD44^hi^) response at the site of infection during *S*. *aureus* challenge, with a prevailing central memory phenotype (Fig 10C). Although a transient increase in local Th17 responses was observed in the immunised group this was not significantly different to control groups (Fig 10D).

Finally, we established the downstream effects of this protective Th1 immunity. While CCL5 production in the peritoneal cavity was slightly increased in the vaccinated mice (Fig 10E), this did not reach statistical significance and notably did not translate to an increase in total macrophage influx to the peritoneal cavity (Fig 10F). Similar to what was observed in the Th1 adoptive transfer studies this Th1-inducing vaccination strategy had no effect on neutrophil recruitment. Importantly, however, the macrophages present at the site of infection displayed increased activation. The number of infiltrating macrophages expressing MHC II was increased in the CpG+ClfA vaccine recipients, suggesting an activated phenotype (Fig 10G). Using 123-dihydrorhodamine staining to measure ROS in a subgroup of mice, significantly elevated respiratory burst activity was seen in macrophages (Fig 10H)–but not in neutrophils (68.6% vs 65.7% for ClfA+CpG vs CpG only groups, respectively)–of vaccine recipients compared to control groups at 72 h post-challenge. Taken together, these results demonstrate that vaccination with ClfA+CpG can drive Th1 responses, which in turn enhance macrophage effector functions to accelerate clearance of *S*. *aureus* infection.

## Discussion

This study demonstrates, for the first time, a correlate of protective immunity in *S*. *aureus* infection in mice that is also evident in human infection. *S*. *aureus* antigen-specific IFNγ-producing CD4^+^ (Th1) cells are expanded in both humans and mice following infection, and these cells are definitively protective in mice. This protection is manifested as a significantly accelerated clearance of bacteria and mediated–at least in part–by enhanced macrophage responses. We have utilised this information in rational vaccine design, and generated a novel *S*. *aureus* vaccine to specifically target memory Th1 cells. Critically important in that process was establishing that our chosen antigen can induce a human Th1 response, and then combining this antigen with a proven Th1-driving adjuvant to enhance anti-*S*. *aureus* Th1 protective immunity.

Immunological memory is induced by *S*. *aureus* exposure during infection or colonisation. Analysis of this memory response has largely focused on *S*. *aureus*-specific antibodies to date, but induction of an antibody response alone may be incapable of mediating full protection against *S*. *aureus* infection. Clinical observations and data from experimental models now imply that cellular memory responses play a prominent role in anti-staphylococcal immunity [13]. While immunological memory may not equate to complete protection against re-infection, it may influence and improve outcome during subsequent infection. A clinical study intriguingly showed that prior exposure to *S*. *aureus* via nasal carriage was associated with a lower all-cause and *S*. *aureus*-attributable mortality in BSI, as compared with non-colonised individuals [38]. The mechanism behind such protection was not identified and whether it might indicate antigen-specific T cell memory and immunomodulation during invasive infection is unknown. We have previously shown that *S*. *aureus* exposure can favourably modulate T cell responses to improve outcomes during later infection. Using a model of recurrent exposure followed by subsequent challenge, we demonstrated that prior exposure to *S*. *aureus* accelerates clearance of infection in murine *S*. *aureus* peritonitis [30]. Acquired immunity in this model was associated with the expansion of a population of memory γδ^+^ T cells exclusively producing IL-17, but no detectable IFNγ. We now demonstrate that prior exposure to *S*. *aureus* also induces an antigen-specific CD4^+^IFNγ^+^ (Th1) memory response that may also contribute to protective memory. Adoptive transfer of *S*. *aureus*-specific Th1 cells conferred significant protection during systemic challenge of naïve mice potentially by enhancing macrophage effector function, leading to improved clearance of bacteria. Macrophage depletion increased mortality and appeared to result in increased bacterial dissemination. Importantly, the protection seen following transfer of *S*. *aureus*-specific Th1 cells occurred in the absence of an enhanced IL-17 response. Furthermore, transfer of *S*. *aureus*-specific Th17 cells failed to confer any significant protection against systemic *S*. *aureus* challenge.

Considering the results of our previously published study [30] together with our current findings, it appears that in the context of *S*. *aureus* systemic peritonitis, protection induced by prior exposure is associated with both type 1 immunity and IL-17^+^γδ^+^ T cell responses. In contrast, during primary infection, an IL-17^+^γδ^+^ T cell response appears to predominate [30], with no detectable IFNγ present in the peritoneal cavities of naïve mice following *S*. *aureus* challenge. Interestingly, expansion of memory IL-17^+^γδ^+^T cells appears to be restricted to the peritoneum and local draining lymph nodes, and was only evident for the first several hours after challenge infection [30]. In contrast, *S*. *aureus* antigen-specific Th1 memory responses were also evident systemically in the spleen, and this local and systemic Th1 expansion was sustained for several days. It is possible that the IL-17 response from tissue-resident γδ^+^ T cells is more localised to the peritoneal mucosa and acts primarily to prevent invasion and dissemination of infection, whereas a Th1 response is critical to control and ensure complete clearance of systemic infection.

Accumulating evidence suggests that an IL-17 response is particularly important for protection against *S*. *aureus* infection at mucosal sites [20]. A recent study demonstrated that a model vaccine driving anti-*S*. *aureus* Th17 (IL-17 and IL-22) immunity effectively contained cutaneous infection and reduced the risk of systemic dissemination [39]. This mucocutaneous site-specific protective role for IL-17 during *S*. *aureus* infection is supported by the clinical picture in patients with IL-17 deficiencies, who present with skin and lung abscesses, but no increase in invasive disease [20]. In these patients, CD4^+^ T cells are documented as the cellular source of IL-17, whereas γδ^+^ T cells have been identified as the predominant source of IL-17 during murine mucosal infections [40,41]. No study as yet has attempted to characterise IL-17-producing γδ^+^ T cells in human *S*. *aureus* infection.

Our current findings identify that memory Th1 cells expanded as a consequence of prior exposure to *S*. *aureus* are key mediators of protection in systemic *S*. *aureus* infection in mice, and importantly this was the overwhelming population detectable during *S*. *aureus* BSI in the human cohort. Murine studies have previously illuminated an important role for Th1 cells in systemic *S*. *aureus* disease as T cell-deficient and IFNγ-deficient mice are hyper-susceptible to bloodstream infection [8,29]. Other *in vivo* studies examining the role of Th1 cells in varied *S*. *aureus* infection models have generated conflicting results–some have identified no role for IFNγ signalling [40], others a detrimental effect [42] and other studies suggest protection [43]. These differences have largely depended on the site of infection and stage of disease [44]. In the context of systemic disease, however, IFNγ-producing Th1 cells appear essential early in the course of infection. Traditionally, type I immunity is associated with protection against intracellular bacteria via IFNγ stimulation of phagocytes to enhance their activation and effector functions [45]. Emerging evidence also demonstrates that *S*. *aureus* can survive intracellularly as a means of immune evasion and dissemination [46,47]. Thus, induction of type I immunity may aid clearance of these intracellular organisms in addition to promoting phagocytosis of extracellular *S*. *aureus*.

Th1 and IL-17^+^γδ^+^ T cell populations may act independently to confer protection during *S*. *aureus* infection, with Th1 activity responsible for eradication of intracellular staphylococci by driving enhanced macrophage responses, and IL-17 activity driving neutrophil recruitment to eliminate extracellular staphylococci. Equally, they may work synergistically, with one pathway triggering or driving the other. Immunoregulatory cross-talk between γδ^+^ T cells and Th2 lymphocytes is recognised [48], although Th1:γδ^+^ interplay is not yet described. Interestingly, inducing combined IFNγ/IL-17 responses (Th1/Th17 in these cases) generated more efficient protection against systemic *S*. *aureus* or pertussis infections than driving a single cell population [29,49]. Significant work beyond the scope of this current study is required to fully characterise the relationship between memory γδ^+^ T cells and memory Th1 cells in conferring protective immunity against *S*. *aureus* infection.

The critical factor that has impeded *S*. *aureus* vaccine development to date has been an absence of data documenting successful translation of findings from mouse models to patients. The suggestion that T cell immunity is central to protection in mice is now widely accepted [13], but there is virtually nothing known about the specific T cell subsets expanded during *S*. *aureus* infection in humans. We have demonstrated for the first time, to our knowledge, that invasive *S*. *aureus* infection in humans predominantly expands a population of circulating *S*. *aureus* antigen-specific memory Th1 cells, suggesting that this population is primed and expanded *in vivo* during infection, and may thus play a role in recovery. *S*. *aureus* antigen-specific Th17 cells were also expanded in SA BSI patients although the memory Th1 population was predominant.

Some limitations of our human cohort include its small size, although the numbers of *S*. *aureus* BSI patients recruited here compares favourably to larger scale multicentre studies with significantly longer recruitment periods [50]. Recruitment of such an acute cohort given the restrictive inclusion criteria, narrow time window from presentation to sample collection and necessity for fresh analysis of cells presented several logistical challenges. The narrow patient eligibility criteria were chosen in an effort to investigate the ‘normal’ T cell response during recovery, and avoid iatrogenic or other confounding effects. A further limitation was that neither pre-morbid colonisation status nor baseline *S*. *aureus*-specific T cell responses of infected patients were known. Humans have ‘prior exposure’ to *S*. *aureus*, which imprints on their adaptive immune system [34]. Our mouse models have determined that such exposure modulates T cell responses to influence the outcome of subsequent infections. It is therefore desirable that ‘pre-morbid’ immunity is taken into account in future studies of vaccines or immunomodulatory therapies. The cohort is also biased towards those SA BSI patients who survive the first 24–48 hours of infection, given that it takes at least this period to confirm the diagnosis. Those patients who present *in extremis* and rapidly deteriorate may have a very different T cell response. While we have demonstrated for the first time, to our knowledge, that *S*. *aureus* antigen-specific memory Th1 responses are expanded in human bloodstream infection, we have not determined whether this is associated with improved patient outcomes. Nonetheless, there are some clinical data to suggest that a pro-inflammatory adaptive cellular response early in the course of systemic *S*. *aureus* infection may be linked to patient survival. Indeed, early and sustained high pro- to anti-inflammatory cytokine ratios predict improved survival and enhanced clearance of *S*. *aureus* bloodstream infection [51]. Despite these difficulties, studies such as ours are vital to advance the knowledge of immune responses during recovery from acute infections in real patients. Future studies can now be designed to correlate the potency of Th1 responses over time with clinical outcomes. It is likely that the most effective immune response will be dynamic over the course of infection–to have the right cells in the right place at the right time for the right duration. We are currently only in the very early stages of understanding this process.

Having demonstrated that *S*. *aureus* antigen-specific Th1 cells are expanded in both mice and humans following systemic infection, we produced a model anti-staphylococcal vaccine by combining the antigen ClfA with the Th1-driving adjuvant CpG. This successfully induced ClfA-specific Th1 cellular immunity and conferred significant protection. Previous studies have demonstrated that immunisation with ClfA conferred antibody-mediated protection in animal models of *S*. *aureus* disease [52,53], and serum anti-ClfA antibody titres were elevated in our patients and other cohorts following *S*. *aureus* bloodstream infection [54]. Importantly, ClfA also appears to have some T cell-activating capacity in animal models [33], while a vaccine based on a structurally similar fungal protein has been shown to activate Th1 and Th17 cells in humans [55]. However, ClfA’s capacity for driving T cell responses in humans has not been fully established. In fact, there are no well-established *S*. *aureus* T cell epitopes [56,57], hence our decision to use heat-killed bacteria as a pool of potential non-secreted antigens. The prevalence of the *clfA* gene in all of our clinical isolates and its retained expression following heat-killing indicated that the antigen-specific T cell response seen in patients could potentially be at least partially attributable to ClfA. Future studies are required to identify other specific staphylococcal antigens recognised by these responding human cells. However, we confirmed that ClfA is an effective T cell antigen with the capacity to induce significant CD4^+^ T cell proliferation and IFNγ production in the majority (>70%) of healthy adults tested.

Anti-staphylococcal vaccines to date have either used alum, which preferentially promotes Th2 and B cells [49], conjugation to toxoid, or no adjuvant. None have shown efficacy in prevention or attenuation of infection, and none have specifically targeted T cells. Adjuvants can be a powerful tool to enhance vaccine efficacy by directing the nature of the immune response, and distinct T cell subsets may be explicitly targeted by careful adjuvant selection [58]. Substitution of CpG for alum in the acellular pertussis vaccine drove an antigen-specific Th1/Th17 instead of a Th2/Th17 response, and this change mediated greater protection against a pertussis challenge [49]. Which T cell population to target, and at which sites, is currently unknown in the context of human *S*. *aureus* infection. Having demonstrated Th1-mediated protective immunity in our animal exposure and adoptive transfer models, and shown that memory Th1 cells are similarly expanded in SA BSI patients, we proposed that Th1 cells may be important in systemic infection. Thus, CpG was chosen for use in our model vaccine. CpG is a TLR9 agonist, which is a potent inducer of Th1 responses and has been put forward as a promising vaccine adjuvant [58,59]. This strategy successfully induced protective type 1 immunity in mice.

Protection associated with ClfA+CpG vaccination induced neutralising antibodies in mice, and the contribution of these humoral responses to protective immunity were not specifically evaluated here. Serum antibody profiling revealed that our patient cohort developed substantially higher antibody titres to secreted toxins than to surface proteins. These anti-toxin antibodies may attenuate disease by binding secreted proteins, and thus should be included as targets in a vaccine. However, we provide compelling evidence that T cell-activating antigens should be a key element of future multivalent vaccines, and demonstrated that vaccination with even a single T cell antigen (in combination with an appropriate adjuvant) can generate protective immunity.

The genetic diversity of *S*. *aureus* strains seen in this cohort is notable and consistent with epidemiological studies that have failed to show that invasive *S*. *aureus* strains have uniform characteristics [60]. A clinically useful vaccine, therefore, must comprise antigens effective against multiple strains. A striking finding of this study was the uniformity of individual patients’ T cell responses to the various reference and clinical *S*. *aureus* strains. Despite the fact that strains represented different clonal complexes–hence varied surface and secreted protein expression [61]–each patient’s T cell proliferative and cytokine responses did not vary significantly across them. Thus, the overall anti-staphylococcal cellular immune response was host-specific and similar across multiple *S*. *aureus* strains, rather than strain-specific. This response firstly suggests recognition of antigens that are highly conserved across strains, which is promising for the discovery of *S*. *aureus* T cell antigens. The ubiquity of ClfA expression seen in this study and its ability to activate T cells in a significant proportion of the population demonstrates this concept effectively. Secondly, this host-specificity adds to compelling clinical evidence that the outcome of human *S*. *aureus* infection may be more dependent on host, rather than microbial factors [62], and in particular, on T cell-mediated immunity [63,64].

Our data strongly supports the notion that protection induced by *S*. *aureus* antigen-specific Th1 cells is mediated by enhanced downstream macrophage responses. Heightened macrophage responses were associated with accelerated clearance of bacteria at multiple systemic sites in prior exposure, adoptive transfer and vaccination models. In contrast, depletion of macrophages significantly impaired the ability of mice to clear *S*. *aureus* infection, and was associated with substantial mortality. The fact that *S*. *aureus* antigen-specific Th1 adoptive transfer did not fully correct survival or bacterial clearance at systemic sites while macrophages remained depleted, supports the idea that these macrophages are essential for Th1 cells to confer protection. However, it is also possible that non-macrophage-dependent mechanisms may contribute to the protection conferred by Th1 cells. In addition to stimulating macrophage microbicidal activity, Th1-derived IFNγ can also upregulate expression of MHC molecules to enhance antigen presentation [45] and promote immunoglobulin isotype switching to high-affinity IgG1 and IgG3 antibodies in humans, or homologous IgG2a antibodies in mice [45,65], which bind avidly to phagocyte Fc receptors, activating complement and augmenting killing. These non-macrophage-dependent mechanisms were not specifically examined in this study. Nonetheless, our combined findings strongly suggest that the role of macrophages in *S*. *aureus* infection may have been previously underappreciated. Macrophages likely play an active role in the direct killing of *S*. *aureus* [35,66] and our preliminary data, and that of others [46] suggest that IFNγ can enhance these effects. However, macrophages may also play an important regulatory role during the immune response to infection. In clodronate-treated mice, the absence of macrophages was associated with excessive neutrophil responses, which may actually play a detrimental role by increasing tissue damage. In addition, it has also been reported that neutrophils can act as intracellular reservoirs for *S*. *aureus* and therefore their numbers must be tightly controlled during *S*. *aureus* infection [42,47]. Macrophages actively promote resolution of inflammation through phagocytosis of apoptotic infected neutrophils [67], while a balance between inflammatory and regulatory macrophages–as opposed to excessive inflammatory macrophages–was associated with protection in a primate model of *E*. *coli* infection [68]. Consequently, we must consider that the increased susceptibility to *S*. *aureus* infection and increased mortality observed following clodronate treatment in our model, may have resulted from the removal of regulatory macrophages. Significant work beyond the scope of this current paper is required to understand the regulatory cross-talk between pro- and anti-inflammatory macrophages and neutrophils during *S*. *aureus* infection.

In this study, despite the significantly enhanced clearance of bacteria observed following Th1 cell adoptive transfer and immunisation with a Th1-inducing vaccine, neutrophil influx to and activity at the site of infection was not affected. In keeping with this, Th17 cells–potential upstream controllers of neutrophil influx–were similarly unaffected, and the Th1-macrophage axis took precedence. Given the propensity of *S*. *aureus* to survive, replicate in and escape from phagocytes, mechanisms that augment phagocyte function represent attractive therapeutic targets [46,47]. Indeed, in CGD patients, addition of IFNγ partially corrects the defect in phagocyte ROS production and restores bactericidal activity *in vitro* [69], and IFNγ therapy has consequently been used successfully as prophylaxis in this cohort [70]. IFNγ treatment of macrophages from healthy individuals also enhances intracellular killing of *S*. *aureus* [46,69]. In the context of an efficacious anti-*S*. *aureus* vaccine, however, it is entirely likely that a contribution from T cells producing both IFNγ and IL-17 will be required to confer maximum protection, but that these responses will need to be tightly regulated. In a recent murine anti-LCMV (lymphocytic choriomeningitis virus) vaccine study, vaccine-induced antigen-specific CD4^+^ T cell responses resulted in catastrophic inflammation and death upon subsequent LCMV infection [71]. However, whether or not such effects might translate to humans is unknown. Differences in human pro- and anti-inflammatory T cell subset distribution, APC ratio, antigen dose, co-receptor expression and, crucially, a lifetime of balanced immune responses to multiple exposures, may account for results unpredicted by pre-clinical work. These knowledge gaps in the dynamics of human cellular immune responses during infection, and their association with clinical outcome, should urgently drive further investigational work in patient cohorts. Recently, a clinical anti-*S*. *aureus* vaccine study in cardiothoracic surgery patients demonstrated increased mortality among vaccine recipients who subsequently developed invasive *S*. *aureus* infection [50]. While this vaccine induced a Th17 response in *S*. *aureus*-naïve mice [72], it appears that impaired T cell responses in a subset of vaccine recipients prior to vaccination may actually have contributed to mortality [73].

The current study significantly advances our understanding of the human cellular immune response to *S*. *aureus*, identifying a subset of IFNγ-producing memory CD4^+^ cells that are expanded during recovery from SA BSI. Furthermore, the analogous murine memory Th1 cells are similarly expanded and attenuate the course of infection following re-challenge with *S*. *aureus*. They are the key responders to a model Th1 vaccine (ClfA+CpG) that also accelerates clearance of systemic infection. Th1 cells thus represent a potentially important and novel target for the rational design of future vaccines against *S*. *aureus* infection.

## Materials and Methods

### Mice

Age (6–12 weeks) and sex matched wild-type C57BL/6 mice were obtained from Harlan UK. IFNγ^-/-^ mice were bred in-house. All mice were housed under specific pathogen-free conditions at the TCD Bioresources facility.

### Patients

Immunocompetent adult inpatients from three tertiary care centres in Dublin with bloodstream infection due to *S*. *aureus* or *E*. *coli* who met defined inclusion criteria (S1 Table) were invited to participate. Onset of symptoms was taken to be onset of bloodstream infection, and admitting physicians took blood cultures when clinically indicated by fever or rigors. Blood was inoculated into aerobic and anaerobic BacT/Alert bottles (Biomérieux, France) and incubated in an automated detection system. Positive isolates were identified as either *S*. *aureus* or *E*. *coli* in accordance with CLSI (Clinical & Laboratory Standards Institute) guidelines. On day 7 ± 2 post-onset of bacteraemia, 40ml venous blood was collected and relevant clinical data recorded. In *S*. *aureus* bacteraemia, symptom onset is acute and typically severe, consequently all patients in this study sought medical attention within 24 h. For *E*.*coli*, presentation is more variable, and our patients presented within 12–48 h after onset of symptoms.

### Bacterial strains

For all human assays four reference *S*. *aureus* strains were used along with each patient’s infecting *S*. *aureus* strain. PS80 and SH1000 are laboratory strains described previously [74,75]. Two invasive clinical strains, one methicillin-sensitive (SJH.MSSA-1; CC7) and one methicillin-resistant (SJH.MRSA-1; CC22) *S*. *aureus* from the collaborating clinical microbiology laboratory were also included to represent currently circulating clones. A clinical *E*. *coli* strain was used as a non-staphylococcal control. Patients’ own *S*. *aureus* isolates were cultivated overnight at 37°C on Columbia blood agar (CBA) from recently-made nutrient agar slopes. Reference strains of *S*. *aureus* and *E*. *coli* were cultivated from frozen stocks in the same way. All bacteria were heat-killed for the human assays. For heat-killing, bacterial suspensions were prepared in sterile PBS at OD_600nm_ of 1.0. CFUs were verified by plating serial dilutions onto CBA. Staphylococcal bacterial suspensions were heat-inactivated at 90°C for 45 minutes and *E*. *coli* suspensions at 70°C for 30 minutes and then washed to remove secreted proteins. Gram staining was performed to confirm that killed bacteria remained intact. Killing was verified by plating on CBA and incubating at 37°C for 5 d. Strains were adjusted to a concentration of 25μg total protein per mL (approximately 1 x 10^8^ CFU/ml) as confirmed with Pierce BCA Protein Assay (Thermo Scientific) kit, and stored at 4°C for up to 4 weeks.

For *in vivo* challenge studies *S*. *aureus* strain PS80 was cultivated from frozen stocks for 24 h at 37°C on Columbia agar supplemented with 2% NaCl. Bacterial suspensions were prepared in sterile PBS and adjusted to 5x10^9^ CFU/ml by OD_600nm_ measurement. CFUs were verified by plating serial dilutions onto Tryptic Soy Agar (TSA).

### *In vivo* models

#### *S*. *aureus* recurrent peritonitis

The recurrent peritonitis model has been previously described [30]. Briefly, mice were exposed to *S*. *aureus* (5x10^8^ CFU) by i.p. injection on days 0, 7 and 14 before being allowed to recover for 21 d, at which point residual inflammation had resolved. In this model, bacterial burden in the peritoneal cavity, kidneys and spleen were reduced to the limit of detection at 7 d post-challenge [30]. On d 35, previously exposed infection-free mice were challenged with *S*. *aureus* i.p. (5x10^8^ CFU), alongside a group of naïve mice. At specific time points post-challenge mice were sacrificed for analysis of systemic infection levels and immune responses. Intra-peritoneal challenge with *S*. *aureus* is routinely used to induce systemic infection in mice [66,76].

Macrophage depletion was achieved by i.p. injection of 200 μl dichloromethylene-bisphosphonate (clodronate)-containing liposomes (1 mg clodronate/mouse), PBS liposomes (clodronateliposomes.com), or PBS alone 24 h prior to challenge with *S*. *aureus* [77]. Mice to be sacrificed at later time points (120 h post-bacterial challenge) received a second dose of clodronate liposomes or PBS 72 h after the initial administration, as monocytes begin to re-enter the circulation from the bone marrow as clodronate clears from the blood [78].

#### Adoptive transfer of *S*. *aureus* antigen-specific Th1 and Th17 cells

Wild type (WT) and IFNγ^-/-^ mice were used to generate *S*. *aureus*-specific Th1 cells and Th17 cells respectively [49]. Mice underwent repeated exposure to *S*. *aureus* as above and, 7 d after the third exposure, total leukocytes were isolated from the peritoneal cavity and cultured *in vitro* with heat-killed *S*. *aureus* (10^5^ CFU/ml) in combination with rIL-12 (10ng/ml) to drive Th1 cells or rIL-1β and rIL-23 (10ng/ml of each) to drive Th17 cells. After 96 h incubation, cell supernatants were removed for cytokine analysis by ELISA. Cells were purified using Pan T cell Isolation Kit II (Miltenyi Biotec) as per the manufacturer’s instructions. Live/dead staining was performed to ensure viability of cells for transfer. Polarised *S*. *aureus* antigen-specific Th1 or Th17 cells or OVA-specific Th1 cells (5x10^6^) were transferred via i.p. injection into naïve mice. To generate OVA-specific T cells, groups of mice were injected once subcutaneously (s.c.) with 50 μg chicken egg ovalbumin (Sigma) in complete Freund’s adjuvant (CFA) containing 4 mg/ml (0.4 mg/mouse) of heat-killed *Mycobacterium tuberculosis* (Chondrex) as previously described [79]. 10 d post-immunisation, draining lymph node cells were harvested and cultured *in vitro* with Ova (200 μg/ml) and rIL-12 (10ng/ml) to drive Th1 differentiation. Control groups received 5x10^6^ purified CD3^+^ cells isolated from the spleen of a naïve mouse. At 3 h post-cell transfer, recipient mice received an i.p. challenge of *S*. *aureus* (5x10^8^ CFU).

#### Immunisation strategy

Recombinant ClfA A domain protein (amino-acids 40–559) was expressed and purified from *E*. *coli* by nickel chelate affinity chromatography as previously described [80]. The CpG oligonucleotide was from Oligos, etc. (Wilsonville, OR, USA). Naïve mice were vaccinated via s.c. injection with CpG alone (50μg/mouse), ClfA alone (1μg/mouse), CpG in combination with ClfA or vehicle (PBS) on d 0, 14 and 28. On d 63 mice were challenged with *S*. *aureus* via i.p. injection (5x10^8^ CFU). Prior to challenge, blood samples were collected for analysis of antigen-specific antibody titres and cells were isolated from the inguinal lymph nodes (ILN) and spleens, re-stimulated with ClfA *in vitro* for 72 h, and secreted cytokines analysed by ELISA.

In all *in vivo* experiments, peritoneal exudate cells (PEC) were isolated from infected mice by lavage of the peritoneal cavity with 2 ml sterile PBS, cell-free supernatants were collected for cytokine analysis by ELISA and PEC cells were analysed by flow cytometry. The draining mediastinal lymph nodes (MLN) were isolated and disrupted over 40 μM filters to obtain single-cell suspensions. Kidneys, liver and spleen were homogenised in 3 ml of sterile PBS. Tissue bacterial burden was established by plating serial dilutions of peritoneal lavage or tissue homogenate on TSA plates for 24 h at 37°C.

### Isolation and stimulation of human T cells

PBMCs from BSI patients were isolated, within 3 h of venepuncture, by density centrifugation of PBS-diluted fresh heparinised blood over a Lymphoprep gradient (Axis-Shield) and labelled with 5μM carboxyfluorescein diacetate succinimidyl ester (CFSE) as previously described [81]. CFSE-labelled PBMCs (4x10^5^/well) were cultured in cRPMI alone (negative control), staphylococcal enterotoxin A (100ng/ml, Sigma [positive control]), heat-killed *E*. *coli* (1μg/ml), and 5 strains (4 reference strains as above and patient’s own infecting strain) of heat-killed *S*. *aureus* at 1μg/ml total protein concentration (approximately 1 x 10^7^ CFU/ml). cRPMI comprised RPMI (Sigma), 10% v/v fetal calf serum (Biosera), 100mM L-glutamine (Gibco) and 100μg/ml penicillin/streptomycin (Gibco). To test the T cell-activating potential of purified ClfA, human PBMCs were isolated from healthy blood donor buffy coats. CD4^+^ cells were purified using CD4^+^ T cell Isolation Kit (Miltenyi Biotec) and CFSE-labelled. PBMCs were gamma-irradiated at 30 Gy with a ^137^Cs source (Gammacell 3000, Best Theratronics). CFSE-labelled CD4^+^ cells (1 x 10^5^) were co-cultured with irradiated PBMCs (1 x 10^5^) in 96-well round-bottomed plates and stimulated with ClfA (5μg/ml) or heat-killed *S*. *aureus* as well as the above controls. Cell culture supernatants were collected on day 4 for cytokine analysis by ELISA and T cell proliferation assessed on day 10, in line with similar studies [82].

### Intracellular staining and flow cytometry

#### Murine experiments

PEC, MLN and spleen cells were incubated in the presence of PMA (50ng/ml), ionomycin (500ng/ml) and Brefeldin A (5μg/ml) (Sigma) for 4 h at 37°C, before surface staining with fluorochrome-conjugated antibodies against CD3 (BD, clone 500A2), γδTCR (Biolegend, clone GL3), CD4 (DB, clone RM4-5), CD8 (eBioscience clone 53-6-7), CD11b (Biolegend, clone M1/70), F4/80 (eBioscience, clone BM8) Ly6G (BD, RB6-8C5), MHCII (eBioscience, clone M5/114.15.2), CD44 (eBioscience, clone IM7), CD62L (eBioscience, clone MEL-14). Cells were fixed and permeabilised using the Dako IntraStain Fixation and Permeabilization Kit, before intracellular staining with fluorochrome-conjugated antibodies against IL-17A (eBioscience, clone 17B7) and IFNγ (eBioscience, clone XMG1.2). Flow cytometric data was acquired with a BD FACSCanto II and analysed using FlowJo software (Tree Star, Inc.). Gates are set on respective Fluorescence Minus One (FMOs). To investigate reactive oxygen species (ROS) activity within phagocytes, 123-dihydrorhodamine assays were carried out as previously described [83].

#### Human experiments

PBMCs or T cell assays were prepared as described above. Brefeldin A (5μg/ml, Sigma) was added to test wells for the final 16 h of culture, while positive control wells (without antigen) were stimulated with PMA (50ng/ml), ionomycin (500ng/ml) and Brefeldin A (5μg/ml) for the final 4 h. Cells were stained for viability with Aqua amine-reactive dye (Invitrogen) before surface staining with fluorochrome-conjugated antibodies against CD3 (eBioscience, clone OKT3), CD4 (eBioscience, clone RPA-T4), CD8 (eBioscience, clone RPA-T8), CD45RO (eBioscience, clone UCHL1), CCR7 (eBioscience, clone 3D12). Cells were fixed and permeabilised using the Dako IntraStain Fixation and Permeabilization Kit, followed by intracellular staining with fluorochrome-conjugated antibodies against IL-17A (eBioscience, clone eBio64DEC17) and IFNγ (eBioscience, clone 4S.B3). Flow cytometric data was acquired with a BD LSRFortessa and analysed using FlowJo software (Tree Star, Inc.). Gates are set on respective Fluorescence Minus One (FMOs). For patient responses, antigen-specific proliferation was calculated by subtracting any background CFSE_lo_ proportions in the negative control wells (i.e. media only) from test CFSE_lo_ populations. Antigen-specific cytokine production in patient samples was similarly calculated by subtracting any background cytokine production seen in negative control wells.

### Antigen-specific humoral responses

IgG antibody titres in mouse blood were quantified by sandwich ELISA as previously described [32]. The presence of ClfA-specific neutralising antibodies was determined by testing the ability of serum from immunised mice to inhibit binding of *S*. *aureus* to fibrinogen. Adherence was calculated as a percentage of adherence in the absence of serum, thus percentage inhibition was determined by subtracting adherence percentage from 100 as previously described [32]. Anti-ClfA antibody titres were quantified in human serum using Luminex bead-based flow cytometry (Luminex Corporation) as previously described [54].

### ELISA

ELISAs for IFNγ, IL-17A, IL-22, TNFα, IL-12p70, IL-23, IL-1β, CXCL1 and CCL5 (R&D Duoset) were performed on murine cell culture or peritoneal exudate supernatants, as per the manufacturer’s instructions. ELISAs for IFNγ and IL-17A (eBioscience) were performed on human serum and human T cell culture supernatants, as per the manufacturer’s instructions.

### Western immunoblotting

*S*. *aureus* SH1000 *clfA clfB*::Em^r^ *fnbA*::Em^r^ *fnbB*::Tet^r^ was described previously [84]. *S*. *aureus* strains were grown in brain heart infusion broth with shaking at 37°C, washed and adjusted to OD_600nm_ of 10. Expression of ClfA was confirmed by Western immunoblotting as previously described [85]. Proteins were separated on 7.5% (w/v) polyacrylamide gels, transferred onto polyvinylidene difluoride membranes (Roche) and blocked in 10% (w/v) skimmed milk proteins. Blots were probed with polyclonal anti-ClfA A domain IgG (1:5,000 or 1:500) and bound antibody detected using horseradish peroxidase-conjugated protein A (1:500, Sigma). Reactive bands were visualised using the LumiGLO reagent and peroxide detection system (Cell Signalling Technology).

### Whole-genome sequencing of *S*. *aureus* isolates

*S*. *aureus* genomic DNA libraries were generated using Nextera XT library preparation reagents (Illumina, Eindhoven, Netherlands) and sequenced on an Illumina MiSeq instrument. Reads were mapped to the *S*. *aureus* TW20 reference genome (NC017331) using the Burrows-Wheeler Aligner (BWA) and nucleotide variant analysis was performed using the SAMtools analysis suite [86].

### Statistical analysis

Statistical analyses were performed using GraphPad Prism software. For murine studies differences between groups were analysed using unpaired Student’s t-tests, Mann-Whitney U tests, one-way ANOVA with Tukey’s comparison post-test or two-way ANOVA with Bonferroni correction post-test where appropriate. For human studies, non-parametric analyses were used unless otherwise stated, as results generally followed a non-normal distribution. Categorical differences between groups were analysed using Fisher’s exact test. Non-categorical differences between groups were analysed using Mann-Whitney U test. Differences between multiple groups were analysed using Kruskal-Wallis test. A p value <0.05 was considered significant.

### Ethics statement

All animal experiments were conducted in accordance with the recommendations and guidelines of the Health Products Regulatory Authority (HPRA), the competent authority in Ireland (project authorisation AE19136/P006) in accordance with protocols approved by Trinity College Animal Research Ethics Committee (License 281109). For the human study, written informed consent was obtained from all patient participants or their next of kin (if patients were incapacitated and unable to give consent). The study was approved by the St James’s and Tallaght Hospitals Joint Research Ethics Committee (2011/35/04) and the Beaumont Hospital Research Ethics Committee (13/101). Anonymised buffy coats were obtained from healthy blood donors at the Irish Blood Transfusion Service, Dublin. Donors have given written approval for the use of cells for scientific purposes.

## Supporting Information

S1 Table Patient inclusion and exclusion criteria. (DOCX)

S2 Table Intra-individual variation in CD4^+^ T cell proliferation to heat-killed *Staphylococcus aureus* strains. (DOCX)

S1 Fig Prior exposure to *S*. *aureus* expands a population of CD4^+^IFNγ^+^ cells but does not affect local innate cytokine production. Groups of mice were exposed to *S*. *aureus* (5x10^8^ CFU) via an i.p. injection on d 0, 7 and 14. Prior exposed mice were then re-challenged with an i.p. injection of *S*. *aureus* (5x10^8^ CFU) on d 35 alongside a control group of naïve mice. At indicated time points post-challenge, cells were isolated from the peritoneal cavity (A) and spleen (B) to assess proportions of IFNγ^+^CD4^+^ and IFNγ^+^CD8^+^cells. At 3 h post-challenge the peritoneal cavity was lavaged with PBS to assess innate cytokine secretion by ELISA (C). Results expressed as mean ± SEM. n = 5 per group. **p<0.005, ***p<0.001.
(TIF)

S2 Fig Transfer of *S*. *aureus* antigen-specific Th1 but not Th17 cells confers protection against subsequent infection and is associated with enhanced macrophage activation. Groups of mice were exposed to *S*. *aureus* (5x10^8^ CFU) via i.p. injections on d 0, 7 and 14. On d 21 the peritoneal cavity was lavaged with PBS and peritoneal cells recovered. A separate group of mice were injected once s.c. with 50μg Ova in CFA and draining lymph node cells harvested 10 d post-immunisation. Cells were polarised *in vitro* with rIL-12 (10ng/ml) and heat-killed *S*. *aureus* (10^5^ CFU/ml) or Ova (200μg/ml) for 96 h at 37°C. Cytokines in cell culture supernatants were analysed by ELISA (A). Groups of mice were transferred 5x10^6^ *S*. *aureus*-specific Th1 cells originating from the peritoneal cavity of previously exposed mice, while control groups received 5x10^6^ Ova-specific Th1 cells or 5x10^6^ naïve splenic CD3^+^ cells, via i.p injection. At 3 h post-transfer all mice were challenged with *S*. *aureus* (5x10^8^ CFU) via i.p. injection. At 72 h post-bacterial challenge, bacterial burden was assessed in the kidneys (B). Results expressed as log_10_ CFU/ml with mean indicated. The total number of MHC II^+^ macrophages (CD11b^+^F4/80^+^Ly6G^-^) present in the peritoneal cavity at 72 h was assessed (C). n = 6–10 per group. At 3 h post-bacterial challenge, the peritoneal cavity was lavaged with PBS to assess IL-17 secretion by ELISA (D). Results expressed as mean ± SEM. Data pooled from 2 independent experiments. CFA = complete Freund’s adjuvant. n = 3 per group. **p<0.005.
(TIF)

S3 Fig Transfer of viable Th17 cells does not protect against subsequent infection. Peritoneal cells were isolated from previously exposed IFNγ^-/-^ mice on d 21 and polarised *in vitro* using rIL-1β and rIL-23 (10ng/ml of each) and heat-killed *S*. *aureus* (10^5^ CFU/ml) for 96 h at 37°C. Cytokines in cell culture supernatants were analysed by ELISA (A). Results expressed as mean ± SEM. 5x10^6^ *S*. *aureus* antigen-specific Th17 cells were transferred to naïve syngeneic hosts, while a control group received 5x10^6^ naïve splenic CD3^+^ cells via i.p injection. At 3 h post-transfer, mice were challenged with *S*. *aureus* (5x10^8^ CFU) via i.p. injection. At 72 h post-bacterial challenge the bacterial burden was assessed in the peritoneal cavity and kidneys (B). Results expressed as log_10_ CFU/ml with mean indicated by bar. Data pooled from 2 independent experiments, n = 8 per group.
(TIF)

S4 Fig CD4^+^ T cell non-specific proliferative responses in patients with bloodstream infection are reduced compared with healthy volunteers and antigen-specific proliferative responses to *E*. *coli* are similar in *S*. *aureus* and *E*.*coli* BSI patients. PBMCs were isolated from healthy volunteers and bloodstream infection patients, CFSE-labelled and incubated with the superantigen staphylococcal enterotoxin A (100ng/ml) (A) or heat-killed *E*.*coli* (1μg/ml) (B) for 10 d before assessing proliferation by gating on CFSE_lo_ CD4^+^ cells using flow cytometry. HV = healthy volunteers; BSI = bloodstream infection. Results expressed as median ± interquartile range. n = 6–17 per group. *p<0.05, ***p<0.001
(TIF)

S5 Fig Invasive clinical and reference strain isolates of *S*. *aureus* display significant genetic diversity. Whole-genome sequencing of invasive *S*. *aureus* clinical (n = 24) and reference laboratory strains (n = 2) was performed and a maximum-likelihood tree is shown. This illustration of genetic diversity is based on 109,533 variant sites identified through comparative analysis of whole-genome sequence data. Branch colours correspond to *S*. *aureus* clonal complex (CC).
(TIF)

S6 Fig Clumping factor A is present in *S*. *aureus* reference and clinical strains and remains present on the cell surface after heat-killing. Cell wall extracts from live *S*. *aureus* were prepared, along with a ClfA-deficient mutant (SH1000 *clfA clfB fnbA fnbB*). Western blots were probed with rabbit anti-ClfA IgG and bound antibody was detected using protein A peroxidase (A). Cell wall extract from heat-killed SH1000 (HK-SH1000) was treated as above (B). The upper band represents full-length ClfA and the lower band a breakdown product.
(TIF)
