# Supplementary material for: Memory Th1 Cells Are Protective in Invasive Staphylococcus aureus Infection
Source: PLoS Pathog. 2015 Nov 5;11(11):e1005226. doi: 10.1371/journal.ppat.1005226 (PMC4634925; doi:10.1371/journal.ppat.1005226)
Supplement: S1 Table — (DOCX) [file ppat.1005226.s001.docx]

**S1 Table. Patient Inclusion and Exclusion Criteria**

| **Inclusion** | - Age ≥18yrs - Confirmed *S. aureus* or *E. coli* bacteraemia, i.e., isolation of a pure organism from ≥ 1 set of blood culture bottles where blood has been collected by use of standard aseptic technique |
| --- | --- |
| **Exclusion** | - Age <18yrs - Mixed bacteraemia - Known active infection with blood-borne viruses:   HIV Ab/Ag positive  Hepatitis C RNA positive  Hepatitis B sAg positive   - Active haematological malignancy - Active solid organ malignancy - Solid organ transplant recipient - Prednisolone therapy >30mg/day for ≥1 month or alternative steroid equivalent - Cytotoxic immunosuppressant therapy:   Calcineurin inhibitors, e.g. cyclosporine, tacrolimus  Antiproliferative agents, e.g. azathioprine, cyclophosphamide, methotrexate, chlorambucil, mycophenylate mofetil  Immune-active monoclonal antibodies, e.g. adalimumab, alemtuzumab, belimumab, golimumab, infliximab, muromonab-CD3, natalizumab, ofatumumab, rituximab, tocilizumab, tocitumomab. |
