# Supplementary material for: Memory Th1 Cells Are Protective in Invasive Staphylococcus aureus Infection
Source: PLoS Pathog. 2015 Nov 5;11(11):e1005226. doi: 10.1371/journal.ppat.1005226 (PMC4634925; doi:10.1371/journal.ppat.1005226)
Supplement: S2 Table — (DOCX) [file ppat.1005226.s002.docx]

**S2 Table.**

**Intra-individual variation in CD4+ T cell proliferation to heat-killed *Staphylococcus aureus* strains**

| Patient | PS80 | SH1000 | SJH.MSSA-1 ^A^ | | SJH.MRSA-1^B^ | Endogenous | *p*-value^C^ |
| --- | --- | --- | --- | --- | --- | --- | --- |
| *S. aureus* BSI | **(%)** | **(%)** | | **(%)** | **(%)** |  |  |
| SA-02 | 38.4 | 0 | | 18 | - | 18^A^ | 0.39 |
| SA-03^1^ | 69.5 | 40.5 | | - | - | 82.3 | 0.37 |
| SA-03^2^ | 9.3 | 3 | | 8.1 | 8.5 | 22 | 0.41 |
| SA-04 | 38.8 | 0 | | - | 43.3^B^ | 43.3^B^ | 0.39 |
| SA-05 | 24.3 | 26 | | 21.2 | 25.9 | 30.5 | 0.41 |
| SA-06 | 5.2 | 0 | | 0 | 0 | 0.6 | 0.41 |
| SA-07 | 47.7 | 6.9 | | 8.9 | 21 | 18.1 | 0.41 |
| SA-08 | 8 | 12 | | 27.6 | 32.5 | 8.1 | 0.41 |
| SA-11 | 8.5 | 4.2 | | 3.9 | 4.3 | 2.7 | 0.41 |
| SA-13 | 23.5 | 13.5 | | 9.8 | 4.8 | 7.1 | 0.41 |
| SA-14 | 5.9 | 3.4 | | 19.4 | 20.3 | 9.7 | 0.41 |
| SA-17 | 3.5 | 1.5 | | 3.1 | 3.3 | 4.2 | 0.41 |
| SA-18 | 0 | 0 | | 0.9 | 0 | 0 | 0.41 |
| SA-19 | 13.8 | 8.2 | | 13.5 | 14.2 | 5.8 | 0.41 |
| SA-20 | 1.1 | 0 | | 0 | 0 | 0 | 0.41 |
| SA-22 | 4.2 | 0.9 | | 2.2 | 2.7 | 2.7 | 0.56 |
| SA-23 | 0 | 0 | | 0 | .72 | 0.38 | 0.41 |
|  |  |  | |  |  |  |  |
|  |  |  | |  |  |  |  |
| ^A^ SJH.MSSA-1 strain was the endogenous infecting strain of patient SA-02 and used as a reference strain for subsequent patients.  ^B^ SJH.MRSA-1 strain was the endogenous infecting strain of patient SA-04 and used as a reference strain for subsequent patients.  ^C^ *P* values are calculated by Kruskal-Wallis test.  ^1,2^ Patient SA-03 had recurrent *S. aureus* BSI (device-associated) 12 months after recovery from initial endovascular infection and was recruited on both occasions.  Endogenous = patient’s own infecting *S. aureus* strain. MSSA = methicillin-sensitive *Staphylococcus aureus*. MRSA = methicillin-resistant *Staphylococcus aureus*. BSI = bloodstream infection. | | | | | | | |
