# Supplementary figures and images for: Memory Th1 Cells Are Protective in Invasive Staphylococcus aureus Infection
Source: PLoS Pathog. 2015 Nov 5;11(11):e1005226. doi: 10.1371/journal.ppat.1005226 (PMC4634925; doi:10.1371/journal.ppat.1005226)

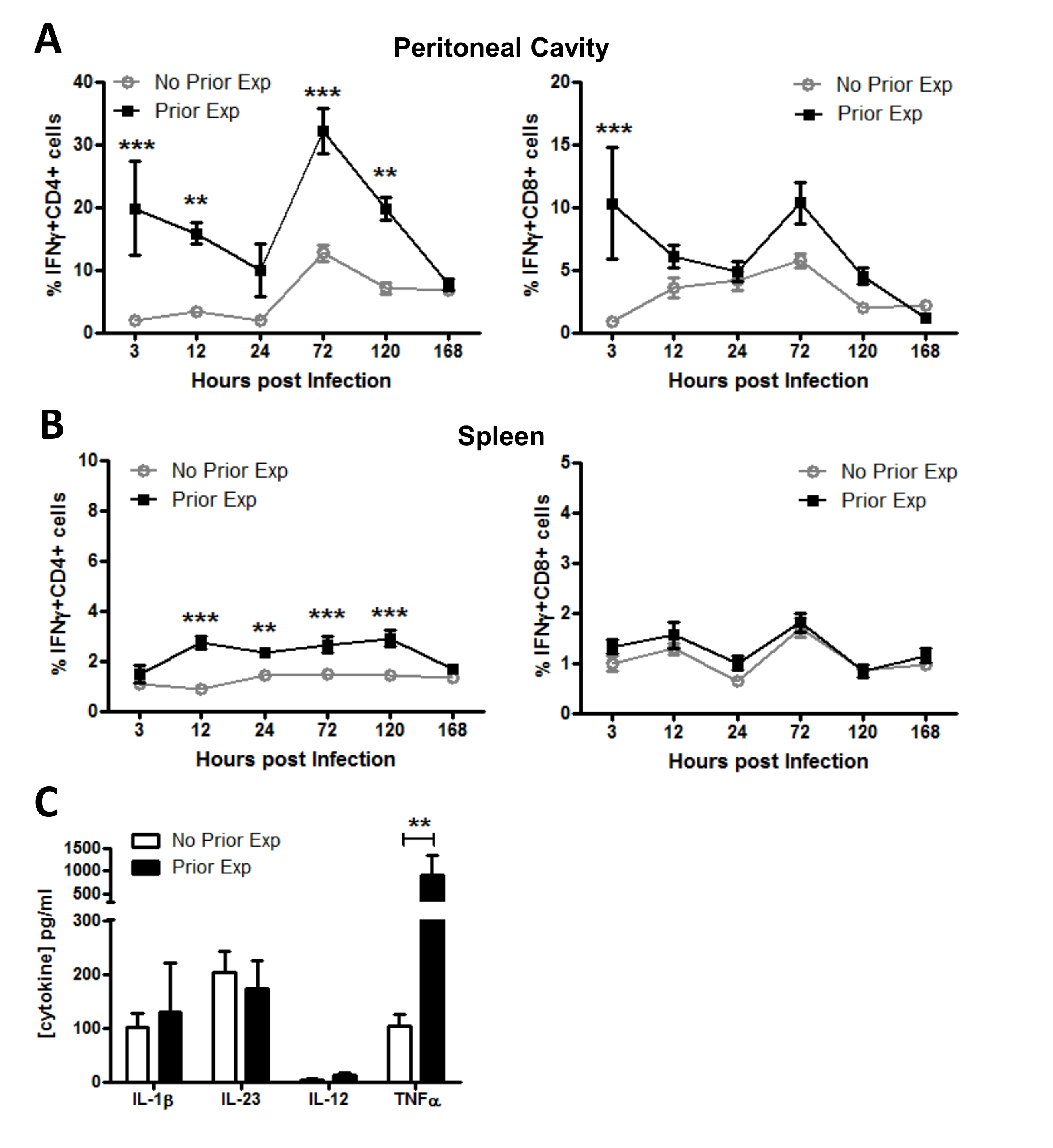

Supplement: S1 Fig — Groups of mice were exposed to S. aureus (5x10^8^ CFU) via an i.p. injection on d 0, 7 and 14. Prior exposed mice were then re-challenged with an i.p. injection of S. aureus (5x10^8^ CFU) on d 35 alongside a control group of naïve mice. At indicated time points post-challenge, cells were isolated from the peritoneal cavity (A) and spleen (B) to assess proportions of IFNγ+CD4+ and IFNγ+CD8+cells. At 3 h post-challenge the peritoneal cavity was lavaged with PBS to assess innate cytokine secretion by ELISA (C). Results expressed as mean ± SEM. n = 5 per group. **p<0.005, ***p<0.001. (TIF) [file ppat.1005226.s003.tif]

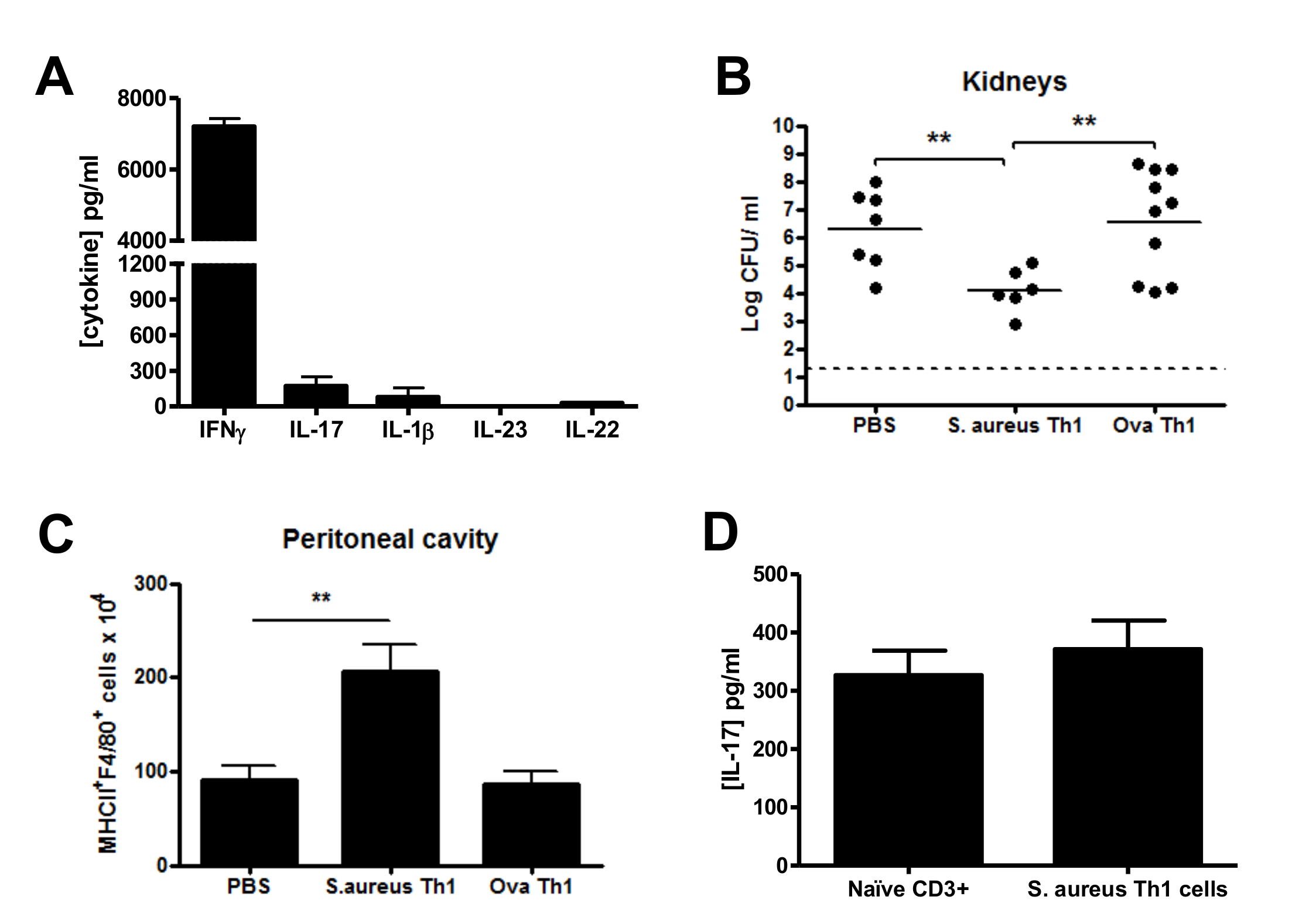

Supplement: S2 Fig — Groups of mice were exposed to S. aureus (5x10^8^ CFU) via i.p. injections on d 0, 7 and 14. On d 21 the peritoneal cavity was lavaged with PBS and peritoneal cells recovered. A separate group of mice were injected once s.c. with 50μg Ova in CFA and draining lymph node cells harvested 10 d post-immunisation. Cells were polarised in vitro with rIL-12 (10ng/ml) and heat-killed S. aureus (105 CFU/ml) or Ova (200μg/ml) for 96 h at 37°C. Cytokines in cell culture supernatants were analysed by ELISA (A). Groups of mice were transferred 5x10^6^ S. aureus-specific Th1 cells originating from the peritoneal cavity of previously exposed mice, while control groups received 5x10^6^ Ova-specific Th1 cells or 5x10^6^ naïve splenic CD3+ cells, via i.p injection. At 3 h post-transfer all mice were challenged with S. aureus (5x10^8^ CFU) via i.p. injection. At 72 h post-bacterial challenge, bacterial burden was assessed in the kidneys (B). Results expressed as log10 CFU/ml with mean indicated. The total number of MHC II+ macrophages (CD11b+F4/80+Ly6G-) present in the peritoneal cavity at 72 h was assessed (C). n = 6–10 per group. At 3 h post-bacterial challenge, the peritoneal cavity was lavaged with PBS to assess IL-17 secretion by ELISA (D). Results expressed as mean ± SEM. Data pooled from 2 independent experiments. CFA = complete Freund’s adjuvant. n = 3 per group. **p<0.005. (TIF) [file ppat.1005226.s004.tif]

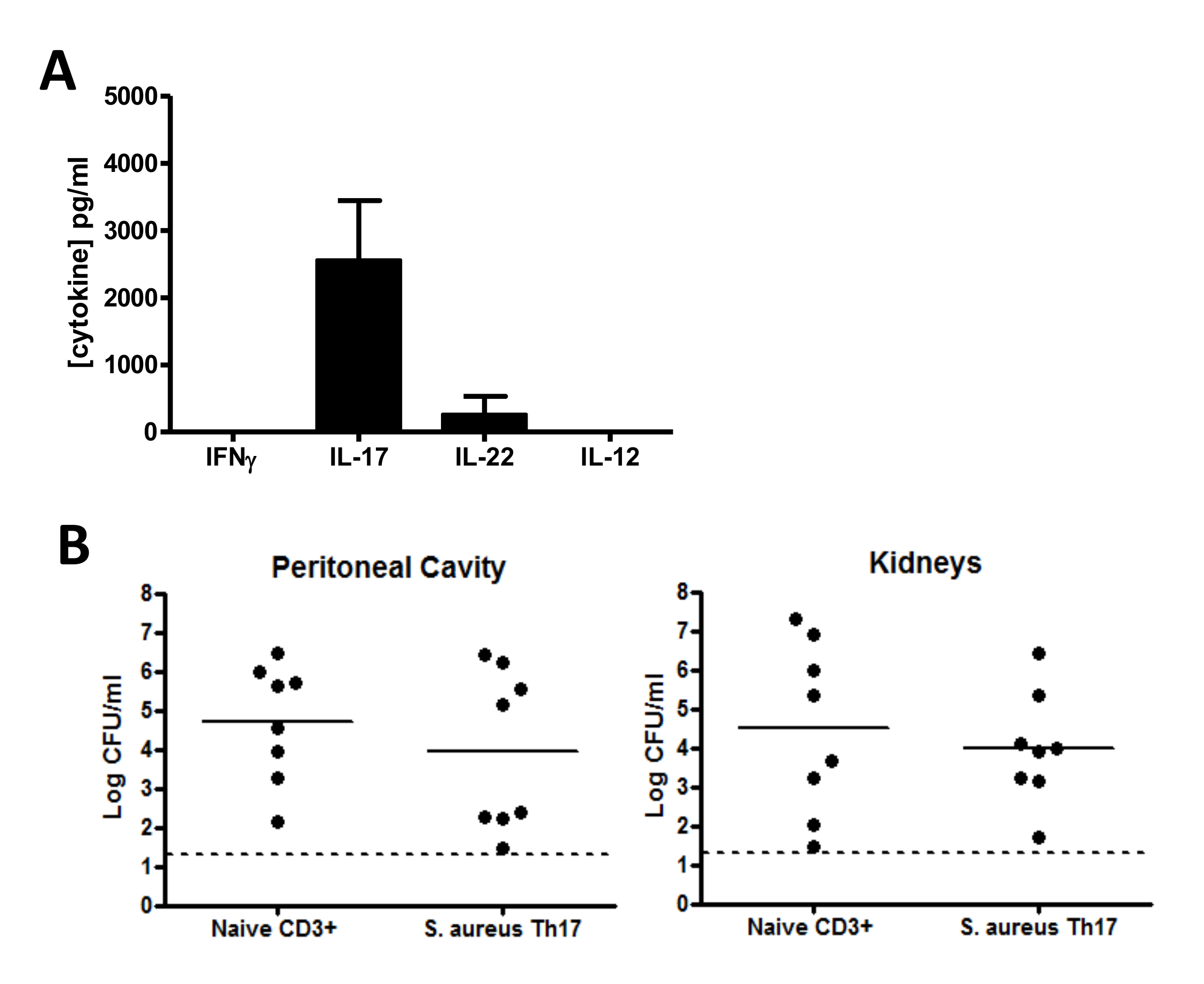

Supplement: S3 Fig — Peritoneal cells were isolated from previously exposed IFNγ-/- mice on d 21 and polarised in vitro using rIL-1β and rIL-23 (10ng/ml of each) and heat-killed S. aureus (105 CFU/ml) for 96 h at 37°C. Cytokines in cell culture supernatants were analysed by ELISA (A). Results expressed as mean ± SEM. 5x10^6^ S. aureus antigen-specific Th17 cells were transferred to naïve syngeneic hosts, while a control group received 5x10^6^ naïve splenic CD3+ cells via i.p injection. At 3 h post-transfer, mice were challenged with S. aureus (5x10^8^ CFU) via i.p. injection. At 72 h post-bacterial challenge the bacterial burden was assessed in the peritoneal cavity and kidneys (B). Results expressed as log10 CFU/ml with mean indicated by bar. Data pooled from 2 independent experiments, n = 8 per group. (TIF) [file ppat.1005226.s005.tif]

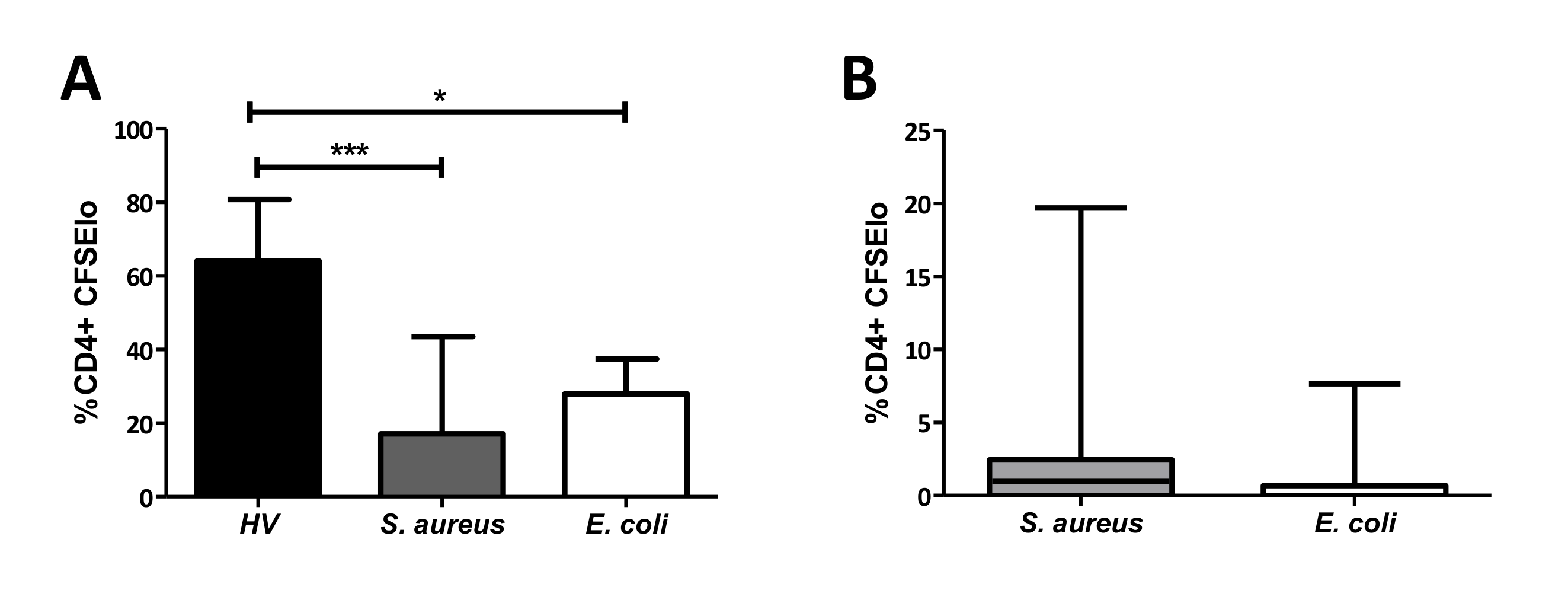

Supplement: S4 Fig — PBMCs were isolated from healthy volunteers and bloodstream infection patients, CFSE-labelled and incubated with the superantigen staphylococcal enterotoxin A (100ng/ml) (A) or heat-killed E.coli (1μg/ml) (B) for 10 d before assessing proliferation by gating on CFSElo CD4+ cells using flow cytometry. HV = healthy volunteers; BSI = bloodstream infection. Results expressed as median ± interquartile range. n = 6–17 per group. *p<0.05, ***p<0.001 (TIF) [file ppat.1005226.s006.tif]

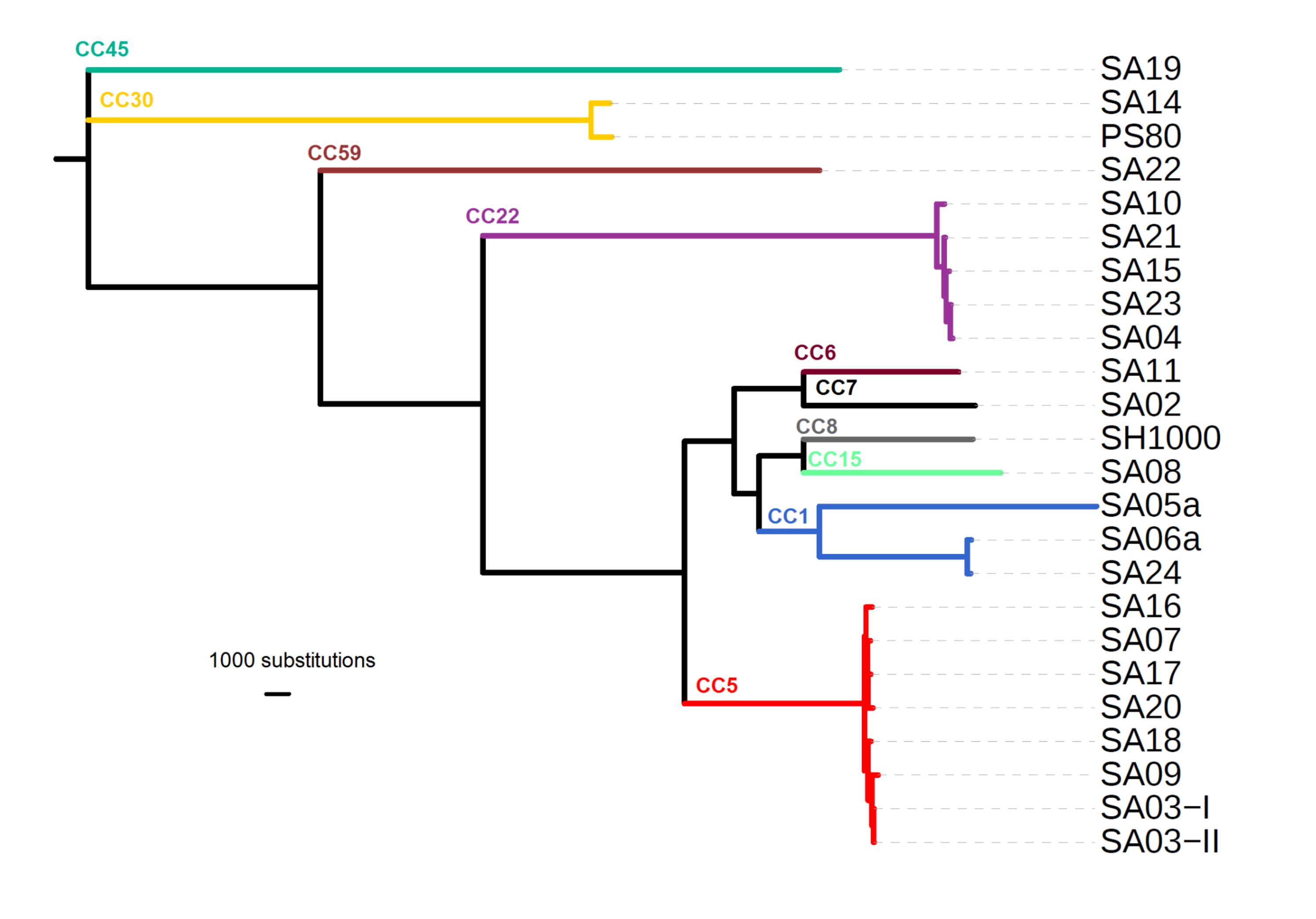

Supplement: S5 Fig — Whole-genome sequencing of invasive S. aureus clinical (n = 24) and reference laboratory strains (n = 2) was performed and a maximum-likelihood tree is shown. This illustration of genetic diversity is based on 109,533 variant sites identified through comparative analysis of whole-genome sequence data. Branch colours correspond to S. aureus clonal complex (CC). (TIF) [file ppat.1005226.s007.tif]

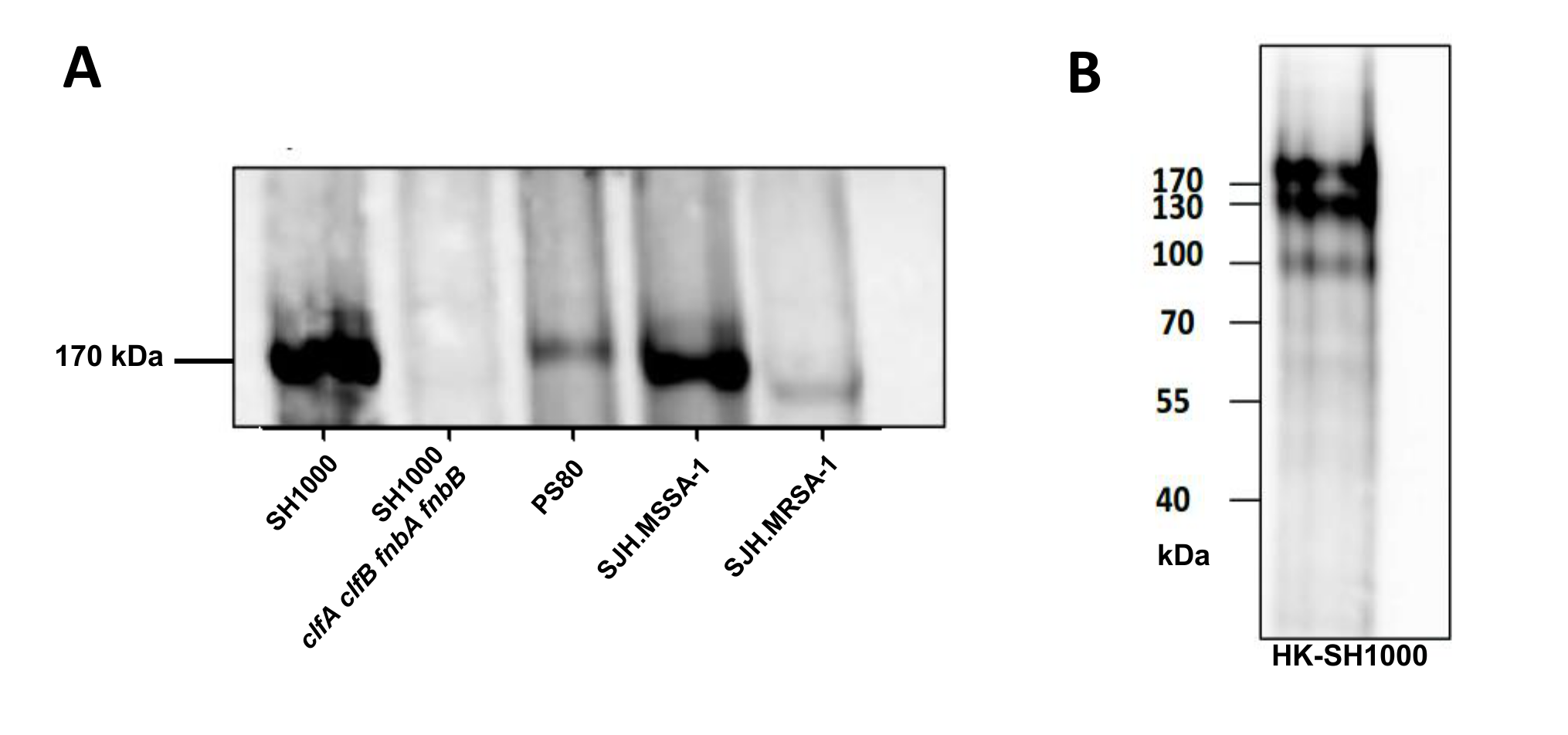

Supplement: S6 Fig — Cell wall extracts from live S. aureus were prepared, along with a ClfA-deficient mutant (SH1000 clfA clfB fnbA fnbB). Western blots were probed with rabbit anti-ClfA IgG and bound antibody was detected using protein A peroxidase (A). Cell wall extract from heat-killed SH1000 (HK-SH1000) was treated as above (B). The upper band represents full-length ClfA and the lower band a breakdown product. (TIF) [file ppat.1005226.s008.tif]
